# Environmental Impacts of Global Offshore Wind Energy Development until 2040

**DOI:** 10.1021/acs.est.2c02183

**Published:** 2022-07-28

**Authors:** Chen Li, José M. Mogollón, Arnold Tukker, Bernhard Steubing

**Affiliations:** † Institute of Environmental Sciences (CML), Leiden University, P.O. Box 9518, 2300 RA Leiden, the Netherlands; ‡ Netherlands Organization for Applied Scientific Research, P.O. Box 96800, 2509 JE Den Haag, the Netherlands

**Keywords:** offshore wind energy, electricity production, prospective life cycle assessment, circularity, scenario analysis

## Abstract

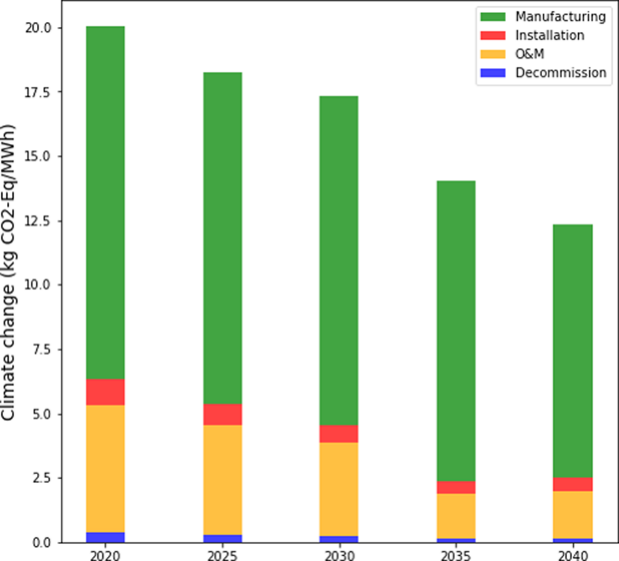

Continuous reduction in the levelized cost of energy is driving the rapid development of offshore wind energy (OWE). It is thus important to evaluate, from an environmental perspective, the implications of expanding OWE capacity on a global scale. Nevertheless, this assessment must take into account various scenarios for the growth of different OWE technologies in the near future. To evaluate the environmental impacts of future OWE development, this paper conducts a prospective life cycle assessment (LCA) including parameterized supply chains with high technology resolution. Results show that OWE-related environmental impacts, including climate change, marine ecotoxicity, marine eutrophication, and metal depletion, are reduced by ∼20% per MWh from 2020 to 2040 due to various developments including size expansion, lifetime extension, and technology innovation. At the global scale, 2.6–3.6 Gt CO_2_ equiv of greenhouse gas emissions are emitted cumulatively due to OWE deployment from 2020 to 2040. The manufacturing of primary raw materials, such as steel and fibers, is the dominant contributor to impacts. Overall, 6–9% of the cumulative OWE-related environmental impacts could be reduced by end-of-life (EoL) recycling and the substitution of raw materials.

## Abstract 2

OWE development will reduce electricity-related environmental impacts and EoL recycling will alleviate raw material requirements and further reduce these impacts.

## 1 Introduction

The installed capacity of global offshore wind energy (OWE) has increased by ∼30% per year from 2000 to 2018. It is furthermore projected to increase 15–24-fold from 23 GW in 2018 to 342–562 GW by 2040.^1^ A key reason for this rapid development is the continuing reduction in the levelized cost of energy.^2^ While OWE development is an effective way to reduce energy-related greenhouse gas (GHG) emissions,^3^ it will incur environmental impacts related to the manufacturing, installation, operation and maintenance (O&M), and decommissioning and end-of-life (EoL) recycling of wind turbines, foundations, and transmission equipment. These impacts remain largely uncertain with the turbine size, the distance from shore both increasing, as well as with recent changes in component technology development.^4^

Life cycle assessment (LCA) studies have shown the significance of lifetime and capacity factors (CFs) for the environmental impacts of electricity generation by the OWE.^5−8^ Region/site-specific studies have been conducted for specific sizes (e.g., ecoinvent 1–3,^7,9−12^ 3–5,^5,13−20^ 8,^21^ and 10 MW),^22^ specific generator technologies (e.g., gearbox based (GB),^7,11−16,19,23^ and direct drive (DD)^22,24^), and specific foundation technologies (e.g., monopile,^7,9,14,17,19,20,23^ gravity-based,^7,13,19^ jacket,^16,19^ and semisubmersible floating^15,18,22,25^). Yet, there is a lack of LCA research quantifying OWE environmental impacts on a global scale that considers future technological developments such as increased turbine sizes and novel component technologies. The evolution of turbine size and market share of technologies have so far not been well considered in the prior studies. The exponential growth of turbine size has promoted increasing CFs in the past years.^26−28^ The average nominal capacity (NC) of commercial turbines increased from approximately 3 MW in 2010 to 6 MW in 2020, and the industry is targeting 15–20 MW turbines in 2030.^1^ Permanent magnet (PM)-based generator technology allows wind turbines to operate at lower speeds and thus have higher efficiencies and energy yields.^29^ Moreover, there are few environmental assessments for emerging technologies, like superconducting generators,^30^ new fiber technologies^31^ in blades, hybrid tower concepts,^32^ and spar, and TLP floating foundations.^33^ Further, potentially important life cycle phases such as the installation, maintenance, and EoL recycling are not well captured in the typical reference life cycle inventory (LCI) data sets (e.g., ecoinvent 1–3 MW, and National Renewable Energy Laboratory (NREL) 5 MW).^34^ Background system changes, such as the energy transition, are also not commonly considered^5,7,9,13−15,17,19,20,23,25^ or not sufficiently and transparently described in the literature.^12,16,18^

This paper develops a prospective LCA model to comprehensively quantify the current and future environmental impacts of OWE development on a global scale until 2040. Dynamic parameterized LCIs were built by including high technology resolution supply chains notably by focusing on installation, O&M, decommissioning, and EoL recycling, which were often neglected in the existing literature. LCIs for a given year (from 2020 to 2040) were adjusted by underlying dynamic trends, including turbine size expanding, moving further from shore, and technology and EoL recycling development. LCA results were furthermore based on futurized background LCI data derived by combining the ecoinvent 3.7.1 database^35^ and information from SSP2 scenarios from the IMAGE-integrated assessment model.^36^ Such an in-depth analysis of the global environmental assessment of the OWE sector allows for a better understanding of the implications of OWE research and development strategies and circular design.

## 2 Methods and Data

**Figure 1:**
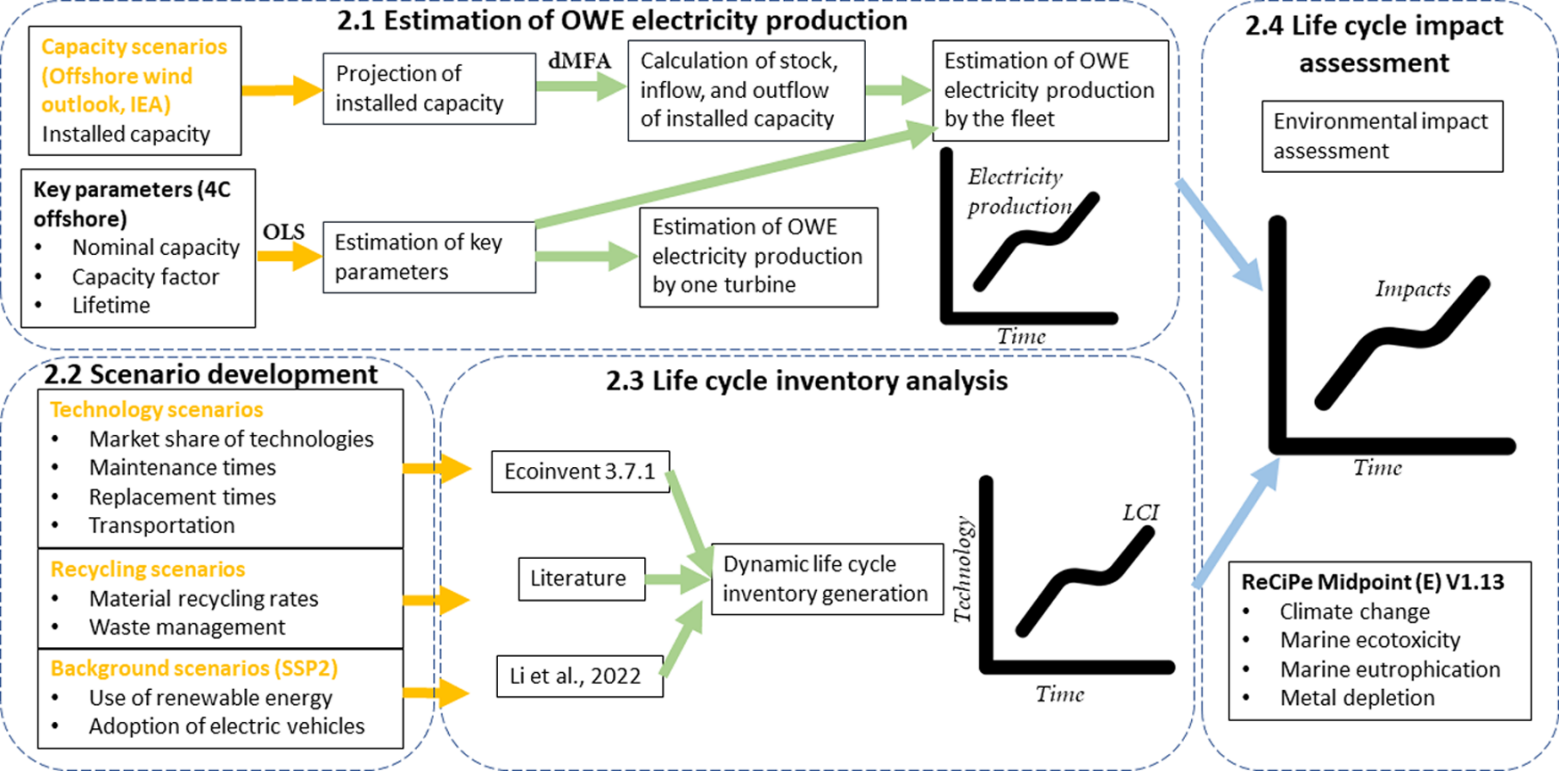
Environmental impact calculation overview.

Environmental impacts were calculated both per MWh and per fleet, spanning from cradle to grave. It thus includes the full life cycle: manufacturing, installation, operation and maintenance (O&M), decommissioning, and EoL recycling. The calculation of environmental impacts is performed via the steps in the subsections below (Figure 1).

### 2.1 Estimation of OWE Electricity Production

The electricity production (EP) by a single wind turbine at year *t* (EP_*t*_^Turbine^) was calculated based on three key parameters, i.e., capacity factor (CF), lifetime, and nominal capacity (NC) (shown in eq 1). The estimation of these three parameters from 2020 to 2040 is introduced in 2.1 in Supporting Information I.
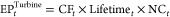
1The EP at the fleet level at year *t* EP_*t*_^Fleet^ was calculated based on CF, lifetime, and installed capacity (stock) (shown in eq 2). The inflow (*I*), stock (*S*), and outflow (*O*) capacity from 2020 to 2040 were calculated based on a dynamic material flow analysis (dMFA)^37^ in line with two IEA OWE capacity scenarios, i.e., stated policy (SP) and sustainable development (SD).^1^
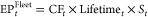
2

### 2.2 Scenario Development

#### 2.2.1 Technology Scenarios

Three technology scenarios, i.e., conventional technology (CT), advanced technology (AT), and new technology (NT), were developed to show technological roadmaps (market shares of technologies in the nacelle, rotor, tower, and foundation)^37^ and applied in this paper. These scenarios were extended by adding maintenance times, replacement rates, and transportation strategies. An overview of the scenarios is presented in Table 1.

**Table 1:** Overview of Preventative (Scheduled) and Corrective (Unscheduled) Maintenance Times, Replacement Rates, and the Transportation Strategy in Conventional Technology (CT), Advanced Technology (AT), and New Technology (NT) Scenarios

technology scenario	technology development	maintenance times (per turbine and year)	replacement rates^1^	transportation means (in addition to workboats being used in near-shore sites)
CT	technology evolution follows a conventional roadmap	two times unscheduled maintenance	high annual replacement rates (∼5%) were assumed as most nacelles are likely to be gearbox based.	no additional
		four times scheduled maintenance (conventional nacelle technologies with high failure rates still dominate the market)		
AT	further development of advanced technologies (e.g., PM-based generators, carbon fibers, hybrid towers, and floating foundations)	two times scheduled maintenance; two times scheduled maintenance (the market brings in more DD nacelle technologies with low failure rates)	moderate replacement rates (from ∼5% in 2020 to ∼3.8% in 2040) were assumed as more DD nacelle technologies with fewer failure rates come to the market.	20% of wind turbines were assumed to be supported by helicopters for sites further from shore with deep waters.
NT	a massive development of advanced technologies, as well as the introduction of new technologies (e.g., PDD and SDD generators, biological fibers, and multiple types of floating foundations)	one time unscheduled maintenance	low replacement rates (from ∼5% in 2020 to ∼3.3% in 2040) were assumed as much more DD nacelle technologies with fewer failure rates are introduced.	50% of wind turbines were assumed to be supported by helicopters for sites further from shore with deep waters.
		one time scheduled maintenance (more DD nacelle technologies with low failure rates are deployed)		

#### 2.2.2 Recycling/EoL Scenarios

Moreover, three EoL scenarios, i.e., hypothetical EoL (EoL_H), optimistic EoL (EoL_O), and conservative EoL (EoL_C), were included in this paper to discuss the environmental performance of EoL second material use and waste material treatment. These scenarios were extended by adding waste management processes, e.g., landfilling or incineration. An overview of these three EoL scenarios is shown in Table 2.

**Table 2:** Hypothetical End-of-Life (EoL) (EoL_H), Optimistic EoL (EoL_O), and Conservative EoL (EoL_C) Scenarios^a^

scenario	recyclable materials	unrecyclable materials	EoL recycling rates	waste treatment
hypothetical scenario (EoL_H)	all	-	all materials from outflow are 100% recycled	no waste materials in this scenario
optimistic scenario (EoL_O)	Fe_L, iron, concrete, Fe_H, Cu, Al, Cr, Mn, Mo, Ni, Zn, B, REEs	polymer (fibers, resin)	bulk materials and key metals were assumed to be recycled with high recycling rates; REEs were considered recyclable; polymer in blades was assumed not recyclable.	most of the waste materials are incinerated; the rest are landfilled
conservative scenario (EoL_C)	Fe_L, iron, concrete, Fe_H, Cu, Al, Cr, Mn, Mo, Ni, Zn	REEs, polymer (fibers, resin)	bulk materials and key metals with low recycling rates were considered recycled; REEs and polymer were assumed not recyclable.	most of the waste materials are landfilled; the rest are incinerated

a Detailed EoL recycling rates can be found in Table S1 in ref (37). Waste treatment processes are introduced in 2.2.4 in Supporting Information I. Materials: Fe_L: low-alloyed steel; Fe_H: high-alloyed steel; and REEs: rare earth elements.

#### 2.2.3 Background Scenarios

Background scenarios are derived from a combination of ecoinvent 3.7.1 data with scenario data of IMAGE. The latter models global future scenarios based on shared socioeconomic pathways (SSPs)^38^ and representative concentration pathways (RCPs). Two “middle-of-the-road” SSP scenarios, SSP2-base and SSP2-RCP2.6,^39^ were derived using the premise framework^40^ and implemented in this study. SSP2-base and SSP2-RCP2.6 showcase the future socioeconomic developments for the years 2020, 2025, 2030, 2035, and 2040, under global warming of 3.5 and 2 °C frameworks, respectively.^41^ We applied linear regression to adapt to each year from 2020 to 2040.

### 2.3 Life Cycle Inventory Analysis

Dynamic parameterized LCIs were generated by including detailed supply chains for state-of-the-art and perspective component technologies in four OWE components, i.e., the nacelle, rotor, tower, and foundation. This was conducted using material flows and stock from a previous paper^37^ and collecting inventories from the literature.^7,13,14,36−38^ LCIs for a given year (from 2020 to 2040) were adjusted by adapting to parameter change (underlying dynamic trends), including turbine size expanding, moving further from shore, and component technology and EoL recycling development. All endpoint supply chains were modeled through the use of ecoinvent 3.7.1 (allocation, cut off by classification).^35^ Lastly, we use the modified LCIs in conjunction with outcomes of the IMAGE for the SSP2-base and SSP2-RCP2.6 background scenarios (Section 2.2.3). The last step leads to a superstructure database that is modeled in the activity browser.^42^ All parameters embedded in the processes are shown in Table S9. An overview of all processes in each life cycle stage is shown in subsections, and a full table that summarizes all processes and corresponding flow values is provided in LCI in Supporting Information II.

#### 2.3.1 Manufacturing

Manufacturing includes processes ranging from material mining, material manufacture, and component fabrication as well as assembly of turbines (including the nacelle, rotor, and tower), foundations, and transmission infrastructures (including cables and transformers). Material requirements from 2020 to 2040 for manufacturing turbine and foundation (an introduction provided in 2.3.1.1 in Supporting Information I) were calculated based on a dynamic material flow analysis (dMFA) from our previous work.^37^ Energy use for material processing, miscellaneous collection, and assembly was taken from ecoinvent^35^ and adjusted by nominal capacity development. The materials used for manufacturing transmission infrastructure were derived from refs^13, 43^ and adjusted by the distance from shore (details can be found in 2.3.1.2 in Supporting Information I).

#### 2.3.2 Installation

Installation processes consist of towing the foundations and turbines out to the site, laying the cabling, and assembling the final units.^44,45^ Installation processes are primarily related to marine vessel operation, in which impacts were calculated based on vessel work time and fuel consumption.^14,46^ Foundation installation processes vary among foundation types with different work times.^46^ Eight foundations^37^ were classified into four types by their installation processes, i.e., foundation type I: gravity-based and high-rise pile cap; type II: monopile; type III: tripot and jacket; and type IV: floating foundations (semisubmersible, spar, and TLP). For type I and II foundations, sour protection is needed before installation setup. Type II and III foundations need driving piles into the seabed, while type IV relies on mooring systems. Processes related to foundations installation were adapted from ref (47); also see 2.3.2.1 in Supporting Information I. Methods and procedures to install turbines (discussion in 2.3.2.2 in Supporting Information I) were already well managed and only marginally different among turbine types.^48^ This paper adopted turbine installation processes from ref (14) and used them for all turbine types. The installation of transmission includes the installation of transformer substations and cables (discussion in 2.3.2.3 in Supporting Information I), which was modeled based on ref (49).

#### 2.3.3 Operations and Maintenance (O&M)

This paper classified O&M processes into two categories, i.e., preventative (scheduled) maintenance and corrective (unscheduled) maintenance (also see 2.3.3 in Supporting Information I). We assumed 1–2 times of preventative maintenance and 1–4 times of corrective maintenance per year per turbine, in line with the literature.^13,50^ In addition, the replacement of large parts (the generator, gearbox if applicable, and blades) and small parts (0.5 *t* low-alloy steel) were included in this paper. The annual replacement rate of the generator was assumed as 2.5% (DD nacelle technologies) and 5% (gearbox-based nacelle technologies) per turbine, respectively. The dynamic change of replacement rates was assumed in line with the market share of DD nacelle technologies. The annual replacement rate of the blades was assumed to be 5% per turbine. The annual replacement rate of small components was assumed as 36.2% per turbine.^13^ These replacement rate values are in line with the literature.^7,13^

#### 2.3.4 Decommissioning

Decommissioning was considered to be the opposite process of installation (detailed processes are provided in 2.3.4 in Supporting Information I).^51^ The time taken for decommissioning processes was assumed 50% less than installation.^52^ This paper assumed that the removal of turbines and foundations is only included after wind farms reaching their lifetimes. Transmission infrastructures, e.g., transformers and cables were assumed to be left in situ.

#### 2.3.5 End-of-Life (EoL) Recycling

EoL recycling and waste treatment were modeled separately from the decommissioning. Recycled materials were assumed to be indefinitely supplied as raw materials for the OWE sector (closed-loop recycling) and nonrecycled materials were assumed to be either landfilled or incinerated. EoL recycling of OWE materials was discussed in line with three recycling scenarios (Table 2). The energy use for EoL recycling was excluded due to a lack of data. Up to 10% of the recyclate for this sector has been reported^53^ but the energy use for recycling is largely uncertain and may widely vary over for different recycling routes. For instance, the energy use of EoL blade recycling technologies (e.g., mechanical, fluidized-bed, and pyrolysis recycling) were reviewed in the literature and varied over one order of magnitude (0.27–30 MJ/kg).^53,54^ These energy requirements vary due to the recycling technology readiness level and waste treatment capacity and policies (e.g., landfill capacity and policies).^55^ Several lab-scale technologies to improve recycling rates have been developed, but they have not reached cost parity with landfilling.^53^ Detailed processes related to secondary materials from EoL recycling and waste treatment (e.g., landfill and incineration) are shown in LCI_EoL in Supporting Information II.

### 2.4 Life Cycle Impact Assessment

The life cycle impact assessment was conducted using the activity browser^56^ according to ReCiPe Midpoint (H) V1.13^57^ impact categories (a full list shown in Table S8). Climate change, marine ecotoxicity, marine eutrophication, and metal depletion were considered as the most relevant impact categories to electricity production by the OWE (discussion can be found in 2.4 in Supporting Information I). The environmental impacts (EIs) of one turbine were calculated as the ratio of its life cycle impacts and its electricity production (EP), where life cycle environmental impacts (LCEIs) were calculated as the sum of the impacts over the lifetime. The EI per MWh in year *t* EI_*t*_^MWh^ was normalized to one MWh by EI per turbine divided by nominal capacity (NC) (shown in eq 3).
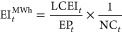
3

Fleet level EI in year *t* (EI_*t*_^Fleet^) was calculated as the sum of the life cycle impact of the wind turbine capacities being part of the fleet and divided by the sum of their EP, where the EI of manufacturing and installation was calculated based on the inflow (*I*) capacities. The EI of O&M and decommissioning (including EoL recycling) was calculated based on stock (*S*) and outflow (*O*) capacities, respectively.^37^
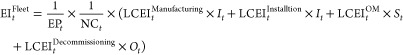
4

### 2.5 Sensitivity Analysis

We performed a sensitivity analysis by modifying parameters by possible ranges, i.e., 20–25 years of lifetime, 50–60% of CF, 5–20 MW of nominal capacity, and 10–100 km of the distance from the shore, to investigate the variances of environmental impacts per MWh based on the AT technology scenario, EoL_O recycling scenario, and SSP2-base background scenario in terms of impact categories considered in this paper. A further sensitivity analysis was performed by altering 20% of several parameters embedded in scenarios, i.e., technology market shares, maintenance time, replacement rates, transportation strategy, recycling rates, and waste treatment.

## 3 Results and Discussion

### 3.1 Environmental Impact Intensity

**Figure 2:**
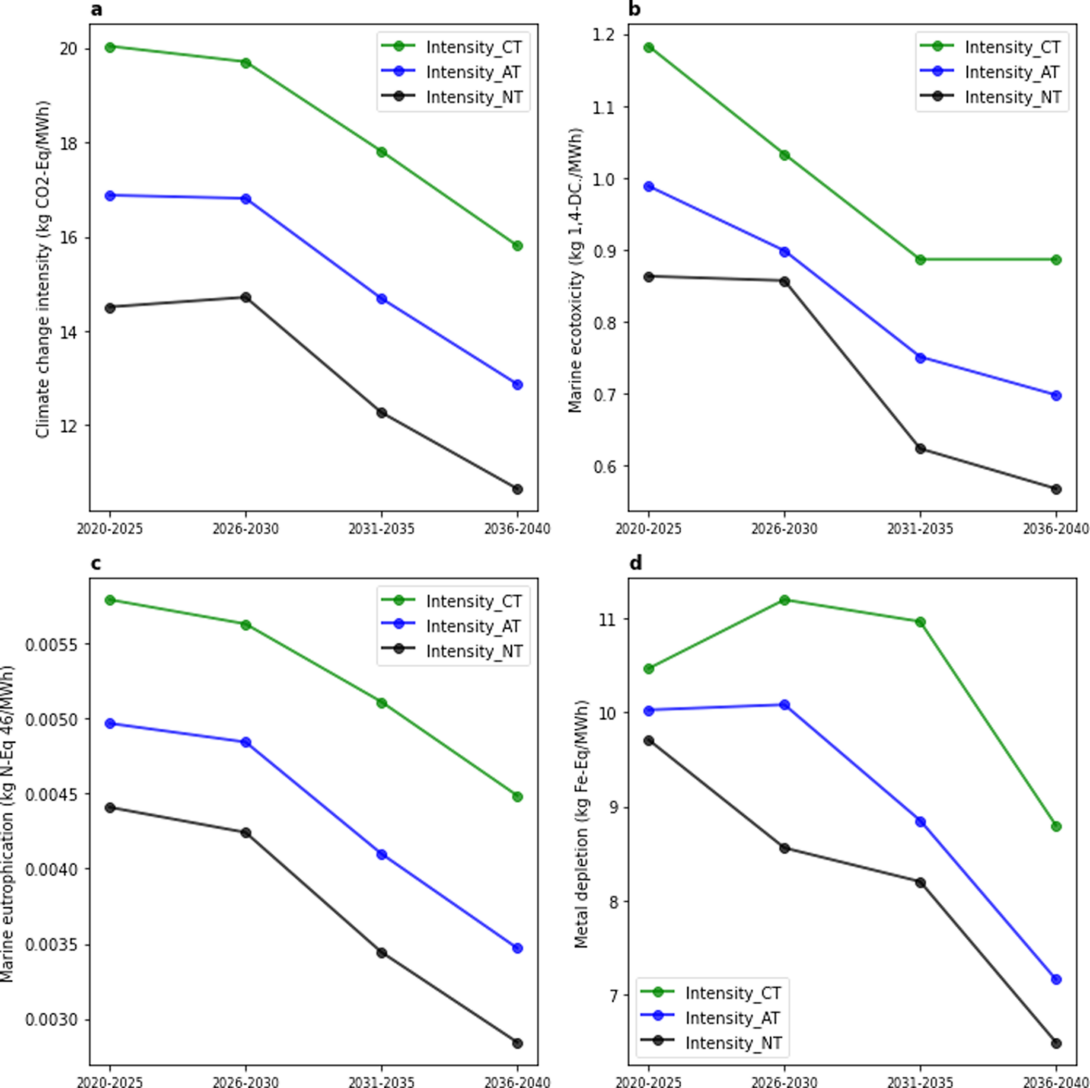
Environmental impacts per MWh, 5 year average, based on conventional technology (CT), advanced technology (AT), and new technology (NT) scenarios, under EoL_O recycling scenario, and SSP2-base background scenario.

The GHG intensity (per MWh) in the CT scenario declines from 20.1 kg CO_2_ equiv for 2020–2025 to 15.8 kg CO_2_ equiv for 2035–2040 (∼21% drop). Similar reductions are found for marine ecotoxicity (∼25% drop), marine eutrophication (∼22% drop), and metal depletion (∼16% drop) (the impact intensity for all evaluated impact categories are shown in Intensity_ReCipe in Supporting Information II). Impact intensities based on AT and NT scenarios are ∼14 and ∼25% lower than in the CT scenario, respectively (Figure 2). This is due to the higher development and market share of advanced technologies in the AT scenario, as well as the introduction of new technologies in the NT scenario. The continuous reduction in environmental impact intensities is expected due to various factors including lifetime extension, size expanding, and technology innovation (3.4, further discussion provided in 3.1 in Supporting Information I).

The environmental impact intensities calculated using the ecoinvent database and NREL 5 MW model result in ∼13.5 and ∼15.0 kg CO_2_ equiv per MWh, which are ∼12 and ∼2% lower than that of the average value (2020–2040) under our baseline scenario AT, respectively. On average, GHG intensities of earlier studies show a much larger variation (7.8–49.4 kg CO_2_ equiv per MWh).^5,7,10,12,14,17−20,58,59^ The variability in these results reflects the differing assumptions, system boundaries, LCI data, and impact assessment methods. In general, our modeled environmental impact intensities are higher than those in refs (12, 17, 19, 55, 57). This may be due to the fact that we included detailed data for installation, O&M, and decommissioning in the life cycle. Further, earlier studies are often constrained by not accounting for underlying dynamic trends, such as changes in turbine size, lifetime, component technology, and recycling development.

### 3.2 Fleet Environmental Impact

**Figure 3:**
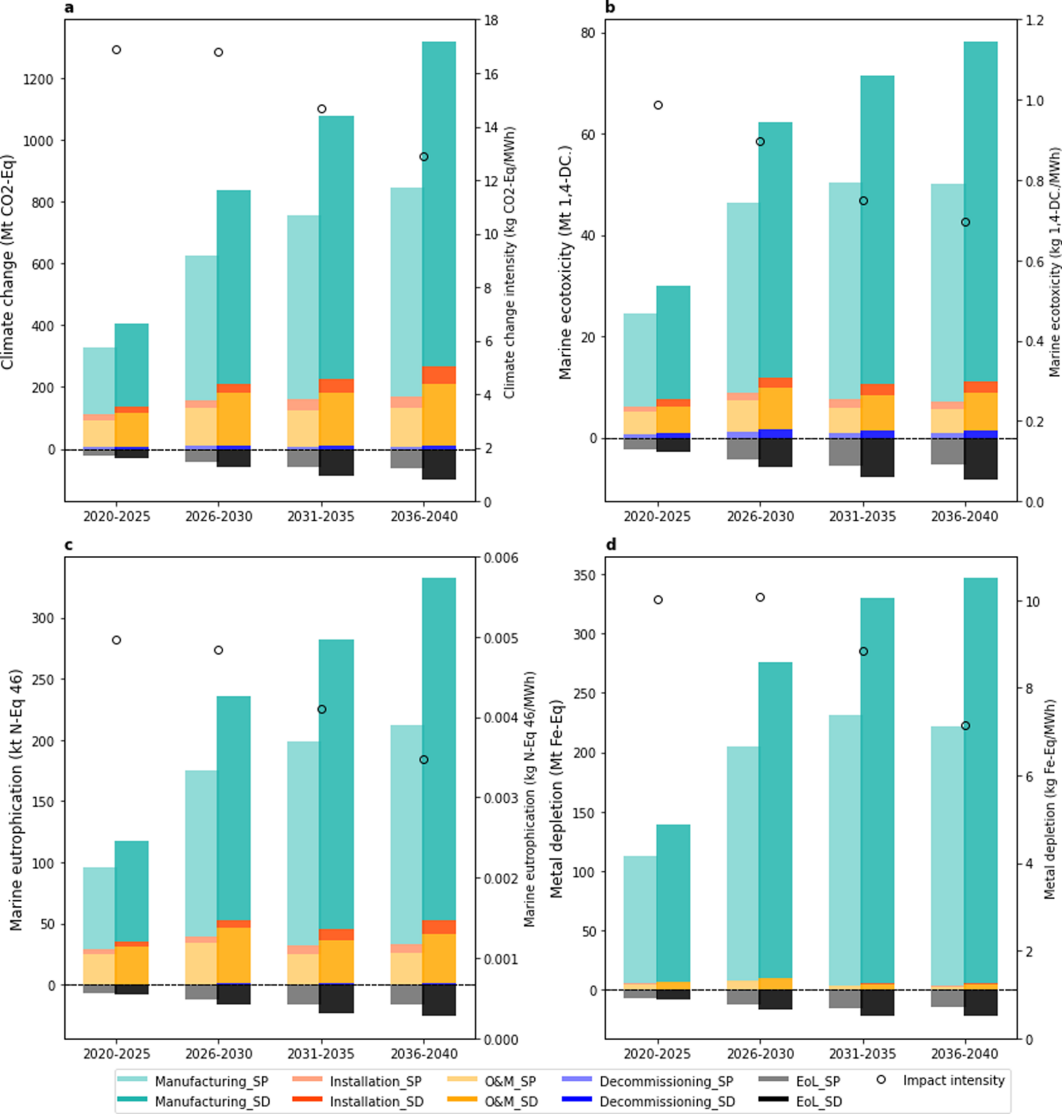
Five-year cumulative fleet environmental impact contribution analysis by the life cycle stage for the stated policy (SP) and sustainable development (SD) capacity scenarios. Five-year average fleet environmental impact intensity (per MWh) for the AD technology scenario, EoL_O recycling scenario, and SSP2-base background scenario.

At the fleet (total installed capacity) scale, the deployment of OWE will cumulatively (2020–2040) result in a contribution of 2.6–3.6 Gt CO_2_ equiv to climate change. However, this compares to 124–207 Gt CO_2_ equiv emissions (48–58 times more) that would be generated when producing the same quantity of electricity with the global electricity mix of 2020. The impacts under the SP scenario are ∼42% lower than that of SD because less installed capacities are assumed in the SP scenario (Figure 3). The deployment of OWE will cumulatively (2020–2040) result in 171 and 242 Mt 1,4-DC to marine ecotoxicity, 0.7 and 1.0 Mt N equiv to marine eutrophication, and 0.8 and 1.1 Gt Fe equiv to metal depletion under SP and SD capacity scenarios, respectively (Figure 3). Fleet impacts on other impact categories are shown in Results_Fleet_Impact in Supporting Information II.

The yearly fleet impacts will increase from 2020 to 2040 as more installed capacities are projected over time. Under the SD capacity scenario, ∼81 Mt CO_2_ equiv climate change-relevant GHG will be emitted from 2020 to 2025. It will increase to ∼168 Mt CO_2_ equiv (∼107% increase) in 2025–2030, ∼216 Mt CO_2_ equiv (∼167% increase) in 2030–2035, and ∼264 Mt CO_2_ equiv (∼226% increase) in 2035–2040 (Figure S9). Such impacts will further increase after 2040 since a substantial new OWE capacity will be installed globally over time.

Renewable energy sources, especially wind and solar power, generated 23.2% of the world’s electricity in 2020 (a 7% increase from 2019).^60^ The increasing deployment of renewable energy systems will result in a large reduction of the environmental impacts of electricity production. According to our analysis (Figure S8), the average (from 2020 to 2040) GHG intensities (per MWh) based on SSP2_base and SSP2-RCP2.6 scenarios are 0.3 (∼2%) and 1.7 (∼10%) kg CO_2_ equiv lower than those of 2020 values, respectively. More importantly, such decarbonization of the electricity system by the OWE has the potential to play an important role by replacing or displacing 408–584 GW of fossil fuels (60.9% of fossil-based electricity),^60^ which would cumulatively (2020–2040) reduce GHG emissions by 124–207 Gt CO_2_ equiv, additional inputs of (essential) metals by 2–3 Gt 1,4-DC, chemical nutrients emissions by 15–21 Mt N equiv, and minerals by 28–34 Gt Fe equiv.

### 3.3 Contribution Analysis

**Figure 4:**
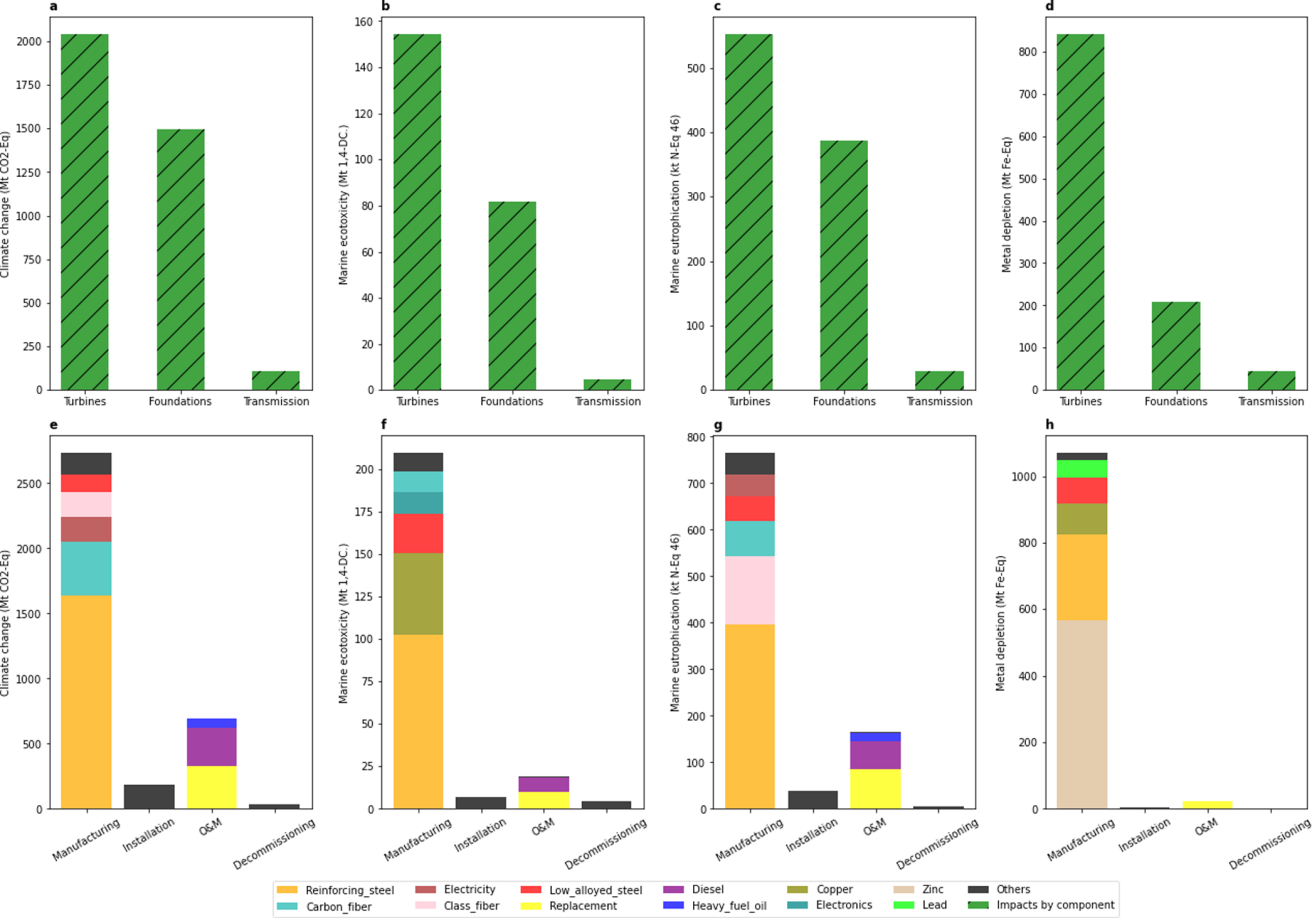
(a–d) Contribution analysis by the component and (e–h) contribution analysis by major processes in each life cycle stage. All contribution analyses were conducted for cumulated (2020–2040) impacts under the SD capacity scenario, AD technology scenario, EoL_O recycling scenario, and SSP2-base background scenario.

At the component level, turbine-related processes (including the nacelle, rotor, and tower) together have the largest cumulative (2020–2040) impacts, i.e., ∼2.0 Gt CO_2_ equiv (∼56%) to climate change, ∼155 Mt 1,4-DC (∼64%) to marine ecotoxicity, ∼0.6 Mt N equiv (∼57%) to marine eutrophication, and ∼0.8 Gt (∼77%) Fe equiv to metal depletion. The foundation-relevant impacts are ∼27 to ∼75% lower than the turbine-relevant impacts (Figure 4 and Table S10). The transmission-relevant processes account for <5% of impacts.

At the process level for each life cycle stage, manufacturing contributes to the largest portion (i.e., ∼75 to ∼98%) of cumulative (2020–2040) environmental impacts, with this contribution increasing from 2020 to 2040 (Figures 3 and S10). This is mainly due to certain raw materials, such as steel (reinforcing steel and low-alloyed steel combined), fibers (carbon fibers and glass fibers combined), copper, and zinc, which are required along with the rapidly growing turbine size and technology development (Figure 4). Steel (reinforcing and low-alloyed combined) has the largest cumulative (2020–2040) contribution among all processes, e.g., ∼1.6 Gt CO_2_ equiv (∼45%) to climate change, ∼104 Mt 1,4-DC (∼43%) to marine ecotoxicity, and ∼0.4 Mt N equiv (∼41%) to marine eutrophication (Figure 4). It is not surprising since a substantial number of supporting structures (e.g., foundations and towers) are made from steel. The fiber in the blade has significant impacts, with ∼17, ∼7, and ∼23% on climate change, marine ecotoxicity, and marine eutrophication, respectively (Figure 4 and Table S10).

Installation contributes ∼5% to climate change, ∼3% to marine ecotoxicity, and ∼4% to marine eutrophication but has a negligible effect on metal depletion (<1%) (Figure 3 and Table S10). The contributions of decommissioning are minor (<2% to all impact categories) as only small portions of turbines (40.1–48.6 GW including turbines installed before 2020 and in the 2020–2040) will be decommissioned between 2020 and 2040. Although installation and decommissioning together account for only small portions of environmental impacts, several processes may cause severe damage to the marine ecosystem.^61,62^ For instance, installation with type II and III foundations (Table S2) may damage the seabed ecosystem due to pile driving. The removal of foundations is furthermore likely to affect the local hard-substrate habitat.^62^ These impacts could likely increase due to more complex installations with wind turbines moving farther from shore into deeper waters. Installation disruptions on the seabed could also be greatly reduced in the near future through the development of the combined turbine-foundation installation technologies.^63^ This innovation will likely reduce installation- and decommission-relevant environmental impacts.^64^

The contributions of O&M to impacts are significant (e.g., ∼0.7 Gt CO_2_ equiv (∼19%) for climate change, ∼19 Mt 1,4-DC (∼8%) for marine ecotoxicity, and ∼0.2 Mt N equiv (∼17%) for marine eutrophication (Figure 4 and Table S10)), but they decrease from 2020 to 2040 (Figure 3). This continuous reduction trend is mainly due to the increasing contribution of manufacturing to impacts, the deployment of DD nacelles with fewer failure rates, and the optimization of marine transportation. O&M impacts are to a large extent underestimated by previous studies (accounts for 1–6% of total global warming potential reported from refs (5, 9, 11)). Our results show that O&M has a relatively high contribution, which is in line with the literature.^13,18,58^ Replacement- and transportation-relevant fuel consumption (diesel and heavy fuel oil) account for the majority of impacts in O&M (Figure 4 and Table S10). These impacts will likely increase due to the higher failure rates related to turbine size enlarging^65,66^ and moving into deeper waters with harsher marine environments.^67^

EoL recycling can alleviate raw material requirements and reduce environmental impacts. Although the vast expansion of the OWE sector implies the inevitable use of primary materials, secondary materials could still represent a substantial source to supply large-scale OWE development. A total of ∼7, ∼11, ∼7, and ∼6% of climate change, marine ecotoxicity, marine eutrophication, and metal depletion can be reduced by EoL recycling, from 2020 to 2040, respectively (Figure 3). Impact reductions by EoL recycling under our hypothetical EoL_H scenario are, based on a 2020–2040 average, around 70% higher than that of under EoL_O scenarios (Figure S11). The contribution of EoL recycling to impact reduction will likely increase as more offshore wind farms reach their EoL after 2030. However, recycling rates are pretty low currently for certain materials, (e.g., fibers and REEs). Impact reductions under the EoL_C scenario (nonrecyclable blades and <1% of REE recycling rates were assumed) are on average (from 2020 to 2040) ∼25% lower than those under EoL_O scenarios, in terms of impact categories considered in this paper (Figure S11). The fiber in blades is currently difficult to recycle, however, and its contribution to manufacturing environmental impact is relatively high, e.g., ∼22, ∼7, and ∼29% of climate change, marine ecotoxicity, and marine eutrophication, respectively (Figure 4 and Table S10). Current methods to address blade waste (e.g., landfilling in pieces and incineration)^68^ are gradually becoming banned due to the costly mechanism and the resulting pollution.^54,55^ According to the European Composites Industry Associate (EUCIA), coprocessing of the fiber in a cement kiln is a viable recycling method for these materials. Mechanical processing of the fiber is under development with high upscaling potential,^69^ with methods under development to improve process efficiency, lifetime, and viable recycling pathways for blades. Further, new technologies have been developed by the OWE industry to enable recyclable blades with organic materials. For instance, Siemens Gamesa has launched fully recyclable offshore wind turbine blades,^70^ and Vestas also aims to make fully recyclable blades by 2030.^44^

REEs are of high economic importance but their production induces high environmental impacts. Less than 1% recycling rates have been reported and few projects have reached desirable scales of REE recycling due to technical challenges.^37^ The industry has strengthened its interest in recovering REEs from OWE facilities and 21% recycling rates are expected.^71^ Greater PM sizes and thus material contents would facilitate the recovery of such magnets and their REEs at the product’s end-of-life stage.^68^ However, meeting sustainability targets will require engagement and collaboration among turbine and blade manufacturers and the cautious consideration of other ethical and environmental impacts.

Although this study assumes that transmission infrastructures will be left in situ, they may one day also be removed. The contribution of transmission infrastructures to impacts is insignificant (<5% for all impact categories, Figure 4) but materials contained in transmission cables (e.g., Cu and Al) have high recycling rates. Steel foundations were assumed to undergo high recycling rates. Overall, ∼3% of climate change-relevant GHG could be reduced by foundation EoL recycling, which accounts for ∼41% of total impacts by EoL recycling (Table S10). However, the removal of foundations is generating controversies due to their extreme costs and potential impacts to the ecosystem.^56,57^ The decommissioning of foundations is likely to leave about a meter of material in the seabed, as opposed to the complete removal.^78^

### 3.4 Sensitivity Analysis

The largest variations of impact intensity are related to turbine size. GHG intensity is doubled (∼225%) and halved (∼58%) when nominal capacity changes from the proposed ones (2.1 in Supporting Information I) to 5 and 20 MW, respectively. The GHG intensity will decrease by ∼11% if the proposed lifetime (from 20 years in 2020 to 25 years in 2040, linear change) alters to 25 years (and increase by ∼11% when the proposed lifetime alters to 20 years). GHG intensity variations of +9 and −9% are observed when the CF is adjusted to 50 and 60%, respectively. The distance from shore variations (which indicates the equipment transportation distance and cable length) has an insignificant effect on the environmental impacts (Figures S12–S15).

Changes in the technology market shares result in a relatively higher level of variability in all impacts than other embedded parameters discussed in this paper. For instance, cumulative GHG emissions from 2020 to 2040 change by approximately +3% (−2%), +4% (−4%), and −9% (+3%) when the market shares of PM-based nacelles, DD-based nacelles, and floating foundation technologies increase (or decrease) by 20%, respectively. Increasing recycling rates and reducing landfill processes by 20% could reduce ∼4 and ∼2% of the environmental impacts. Maintenance times, replacement rates, and transportation strategy have negligible effects on environmental performance. Overall, <2% of variations are seen for all impact categories considered in this paper (Table S11).

### 3.5 Limitations and Outlook

This study evaluated the environmental impacts of global OWE development using a prospective LCA model with various scenarios for technology and EoL recycling development. This LCA model is dynamic at the level of inventory analysis, yet we have not included time-dependent characterization factors as developed, e.g., by Lan and Yao^72^ for greenhouse gases. Activity Browser software did not allow for a dynamic impact assessment. Furthermore, dynamic characterization factors were not available for all impact categories considered. Although the LCIs and results presented here provide new insights at the global level, more specific data and scientific understanding would be still required to adapt the system to regional/local cases. This could be achieved using GIS-based data sets with a higher geographical resolution for site-specific parameters (e.g., wind speed and water depth). Furthermore, ecoinvent processes may underestimate the EI, which could be improved with LCI databases that better represent the downstream supply chain process. For example, small processes are hard to quantify, some important processes are outside of the defined system boundary, and some processes might have high impacts but are even not included in ecoinvent (e.g., production of dysprosium, terbium, and yttrium, and the availability of new materials and specialized vessels).

This study used prospective LCI databases derived from a coupling of the ecoinvent database with data from IMAGE. However, IMAGE is conservative on renewable energy development, (e.g., ∼3.7 and ∼5.3% annual average (from 2020 to 2040) growth of wind energy based on SSP2-base and SSP2-RCP2.6, respectively).^38^ It may lead to a relatively small overestimation of impacts as the background production of materials and supply of electricity do not profit from rapid OWE development. This limitation could be improved by implementing foreground OWE scenarios in IMAGE to assure full consistency between the models. Further, the upscaling of OWE requires larger and more specialized background infrastructure (e.g., uploading and transportation equipment), which is not well modeled (or even unavailable) in the current LCI database, e.g., ecoinvent, (more discussion in 3.5 in Supporting Information I). This study did not include these background infrastructure changes due to a lack of data. Future studies are warranted to consider these background changes and associated impacts. Such improvement would be beneficial to understanding (marginal) cause–effects and feedback mechanisms of OWE technology development using consequential LCA models.

In addition, research is just beginning to unravel the impacts of the OWE on marine ecosystems (e.g., seabed destruction, acoustic disturbance produced by turbine operation and vessel transportation,^73,74^ creation of hard-bottom habitat,^75^ and electromagnetic fields enhancement by underwater transmission cables).^75,78^ Further research is needed to integrate these impacts into a cumulative framework that includes the impact categories considered in this paper. Further uncertainty analyses based on global sensitivity approaches (e.g., Monte Carlo simulation and resampling methods) could be applied in the future to globally assess uncertainties and provide more extensive recommendations.

## Supporting Information Available

More detailed documentation of methods and additional figures and tables that support the modeling and result interpretation (PDF)
Information of parameters, tables of processes and corresponding flow values, and additional results of impact assessment (XLSX)
