# Supplementary material for: Environmental Impacts of Global Offshore Wind Energy Development until 2040
Source: Environ Sci Technol. 2022 Jul 28;56(16):11567–77. doi: 10.1021/acs.est.2c02183 (PMC9386896; doi:10.1021/acs.est.2c02183)
Supplement: Supplementary file 1 — es2c02183_si_001.pdf [file es2c02183_si_001.pdf]

Supporting Information I

Environmental impacts of global offshore wind energy development until 2040

Chen Li[1]*, José M. Mogollón[1], Arnold Tukker[1,2], Bernhard Steubing[1]

[1] Institute of Environmental Sciences (CML), Leiden University, P.O. Box 9518, 2300 RA Leiden, the Netherlands
[2] Netherlands Organization for Applied Scientific Research, P.O. Box 96800, 2509 JE Den Haag, the Netherlands
* Corresponding author: c.li@cml.leidenuniv.nl

Summary information:

The number of pages: 32

Figures: S1-S15

Tables: S1-S11

List of abbreviations

**AT**	Advanced technology	**LCA**	Life cycle assessment
**CF**	Capacity factor	**LCEI**	Life cycle environmental impacts
**CT**	Conventional technology	**LCI**	Life cycle inventory
**DD**	Direct-drive	**NC**	Nominal capacity
**dMFA**	Dynamic material flow analysis	**NREL**	National Renewable Energy Laboratory
**EI**	Environmental impact	**NT**	New technology
**EII**	Environmental impact intensity	**O**	Outflow
**EoL**	End of life	**O&M**	Operation and maintenance
**EoL_C**	Conservative EoL recycling scenario	**OWE**	Offshore wind energy
**EoL_H**	Hypnotical EoL recycling scenario	**PM**	Permanent magnet
**EoL_O**	Optimistic EoL recycling scenario	**PMSG-DD**	Permanent Magnet Synchronous Generator Direct-drive
**EP**	Electricity production	**PMSG-GB**	Permanent Magnet Synchronous Generator gearbox based
**EUCIA**	European Composites Industry Associate	**RCP**	Representative concentration pathway
**FU**	Functional unit	**S**	Stock
**GB**	Gearbox based	**SD**	Sustainable development
**GHG**	Greenhouse gas	**SP**	Stated Policies
**I**	Inflow	**SSP**	Socio-economic pathway
**IEA**	International energy agency		

2. Methods and data

2.1 Estimation of OWE electricity production

The OWE electricity production was calculated based on three key parameters, i.e. capacity factor (CF), lifetime, and nominal capacity (NC). Multiple CFs ranging from 28-60% in the year range 2009-2020 were reported in the literature [1–9] depending on site characteristics (e.g. wind resources) and turbine technology (e.g. gearbox-based or direct drive nacelles). CF is expected to increase as larger wind turbine moving further from shore with better wind resources. Multiply component technology enhancement (e.g. use of permanent magnet-based and direct drive nacelles) will largely increase CF [10–12]. For simplicity, the medium value was used as the estimation of current (in 2020) CF and the maximum value was applied for the expected CF in 2040. This paper assumed dynamic CFs with 50% in 2020 that linearly increases to 60% in 2040. The designed lifetime of offshore wind turbines was estimated to be 20 to 25 years [13]. We applied dynamic lifetimes with a 20-year mean in 2020 that

increases to a 25-year mean in 2040, and a 5-year standard deviation Normal distribution, which is in line with our previous paper [14]. Ordinary least squares (OLS) regression was used to model future NC projections based on existed projects from 4C offshore [15]. More information on lifetime and NC modeling could be found in **2.2** and **2.4.1** in our previous research [14], respectively. **Figures S1** and **S2** show the estimation of CF and nominal capacity, and lifetime, respectively.

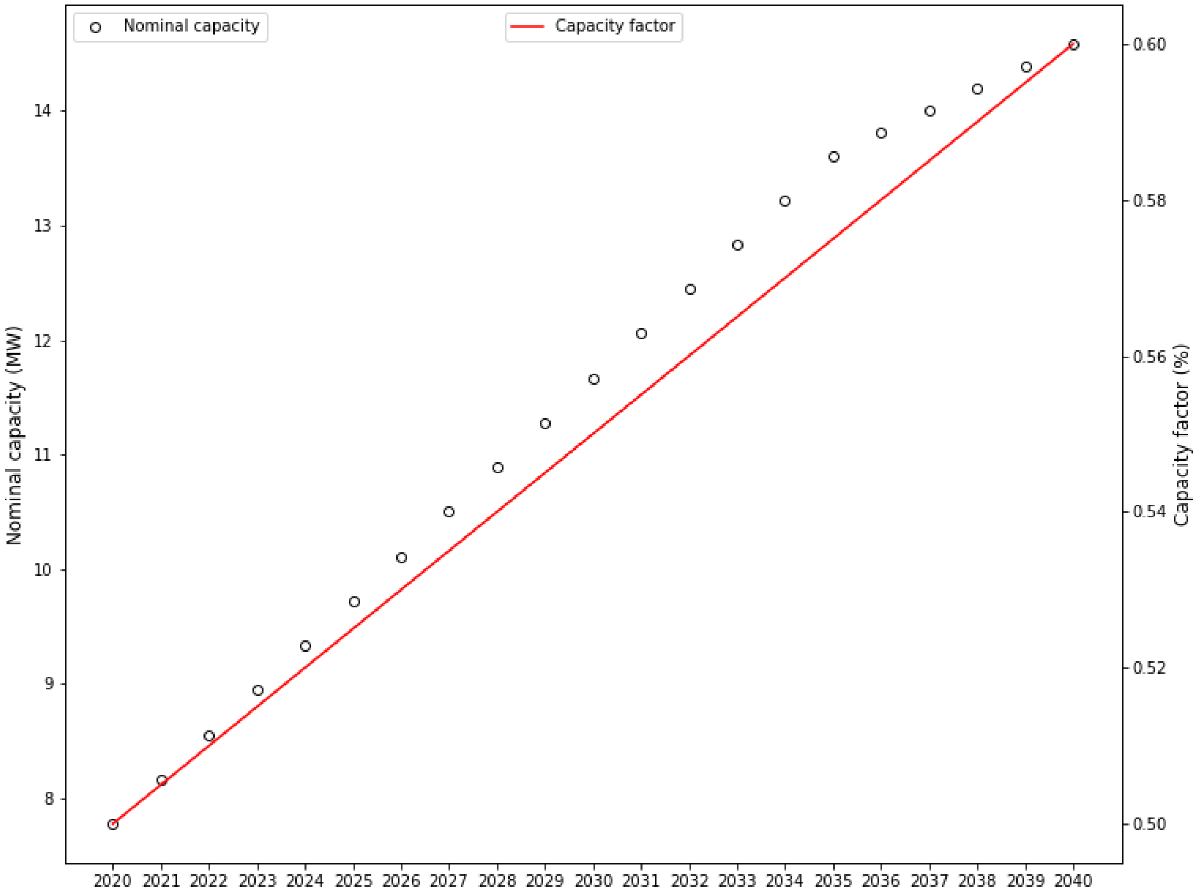

Figure S1: Capacity factor and nominal capacity development from 2020 to 2040

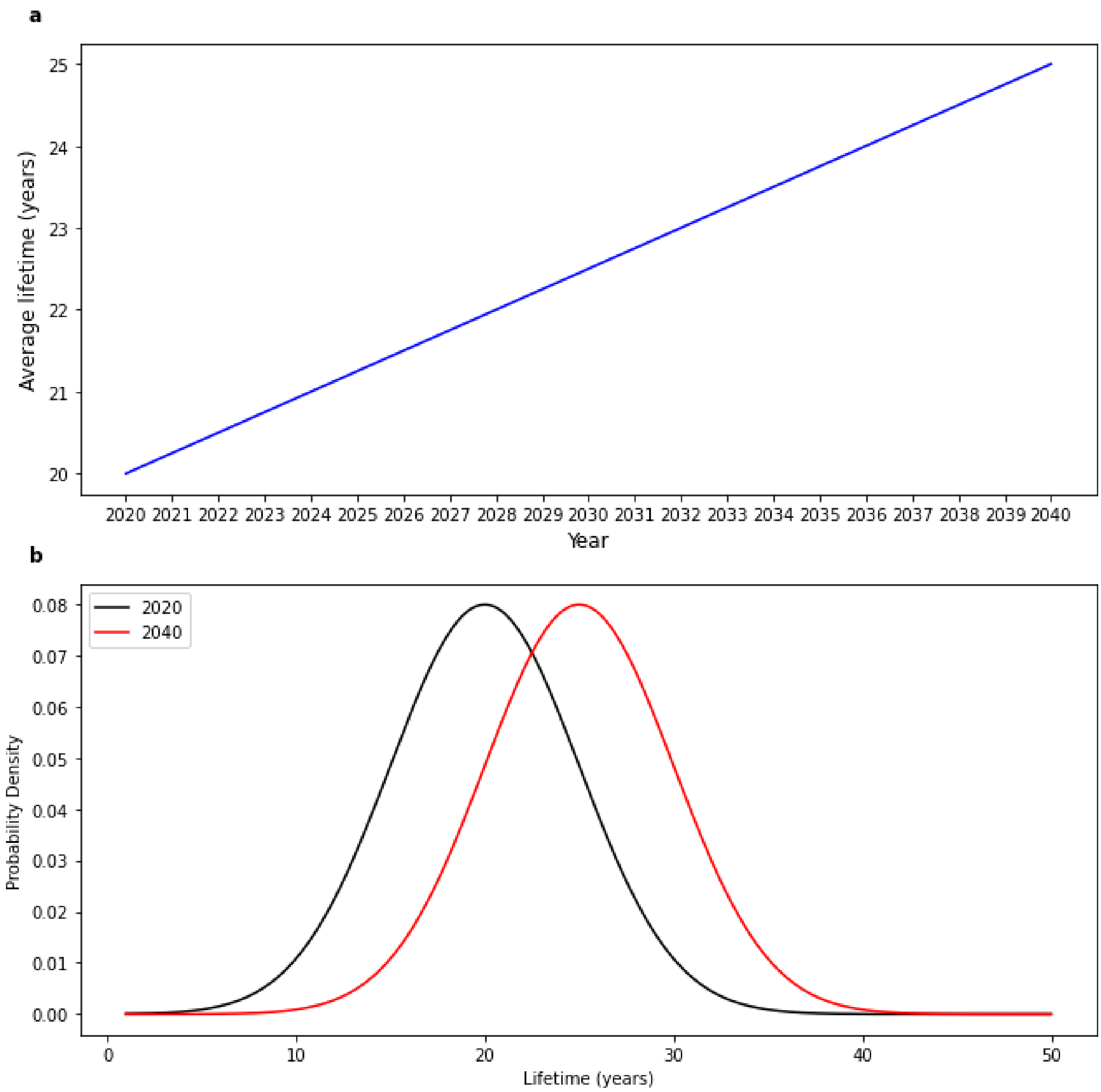

Figure S2: Turbine lifetime development from 2020 to 2040

2.3 Life cycle inventory analysis

Dynamic parameterized life cycle inventories were generated in this paper. Besides key parameters, i.e. CF, lifetime, and NC, turbine size (including rotor diameter and hub height) and distance from shore are another two main parameters. Several processes were adjusted by these two parameters (details provided in **Para** in **Supporting Information II**). OLS regression was used to model the future projections of turbine size based on existed projects from 4C offshore [15] (**Figure 4** in [14]). Future average distance from shore was estimated based on Fraunhofer IEE [16] (**Figure S3**).

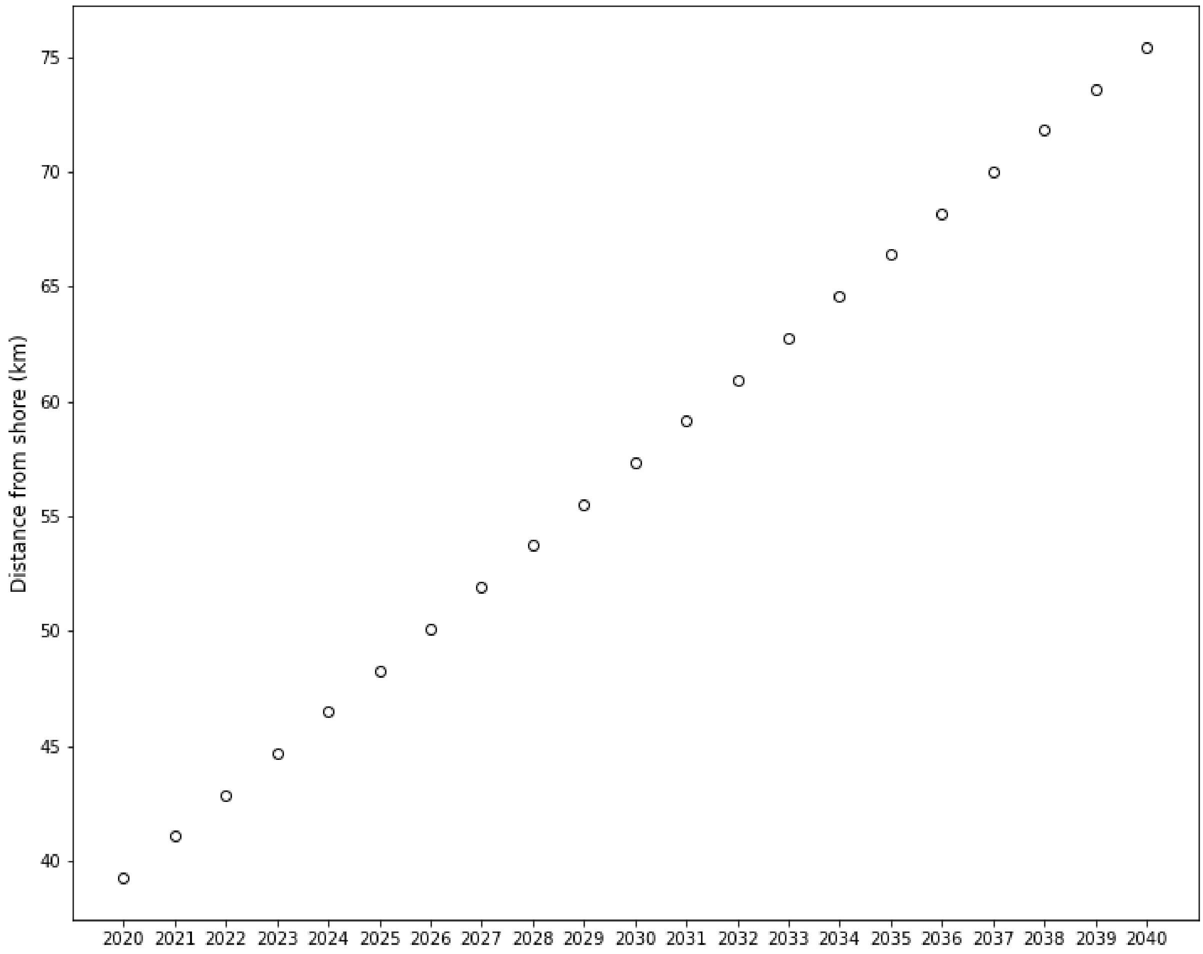

Figure S3: Estimation of average distance from shore.

2.3.1 Manufacturing

2.3.1.1 Manufacturing of turbines and foundations

The material requirements for manufacturing turbines and foundations from 2020 to 2040 were calculated based on the dynamic material flow analysis (dMFA) [14] and used in this paper. A wide assortment of materials was considered, which including bulk materials, rare earth elements (REEs), key metals, and other materials for manufacturing 24 component technologies in the nacelle, rotor, tower, and foundation.

2.3.1.2 Manufacturing of transmission

Material use for manufacturing offshore wind transmission (cables and substation infrastructures) was calculated based on the following assumptions and estimation. Internal cable material requirements were calculated based on cable length and material intensity. Internal cable length was determined by turbine layout. The spacing between turbines in a column would be 5 to 10 times of rotor diameters and spacing between columns would be 7 to 12 times of rotor diameters [17]. As turbine size grows, the spacing is likely to increase. Therefore, the upper bounds were used in this paper. Five types of internal cables (i.e. 3x95 mm2 Cu, 3x150 mm2 Cu, 3x240 mm2 Cu, 3x400 mm2 Cu, 3x630 mm2 Cu) were found currently in the market. In simplicity, the average material intensity value of these five types was used in this paper (shown in **Table S1**). External cables consist of submarine cables, onshore aerial and underground cables. Due to lacking of data and ignorable length, onshore aerial and underground cables were excluded in this paper. Submarine cable length was determined by distance from shore. We considered submarine cable length equal to distance

from shore. External cable material intensity (shown in **Table S1**) has been derived from [9]. A substation was assumed to consist of two ABB's transformers and one foundation per wind turbine. Data of the mass of materials and energy use were derived from ABB's report of Environmental Product Declaration [18]. Substation foundation was assumed to be identical to one fix-bottom based (monopile) foundation.

Table S1: Material intensity (t/km) of cables. * The average of abovementioned five types of internal cables [9].

	Total weight (t/km)	Conductor (copper)	Insulation (polyethelene)	Lead sheath	Galvanized steel	Outer layer (polypropylene)
Internal submarine cables (33kv)*	26.4	22.6%	6.2%	26.2%	41%	4.2%
External submarine cables (132kv)	74	27%	8.7%	25%	35%	4.9%

2.3.2 Installation

Installation of turbine and transmission is with less technological spectrum but the installation processes vary significantly among foundation types [2].

2.3.2.1 Installation of foundation

The foundation installation processes vary among foundation types. Eight foundations [14] were classified into four types by their installation processes, i.e. foundation type I: Gravity-Base and High-Rise Pile Cap; type II: Monopile; type III: tripot and Jacket; Type IV: floating foundations (Semi-Submersible, Spar and TLP). For type I and II foundations, sour protection is needed before installation setup. Type II and III foundations need driving piles into the seabed. While type IV relies on mooring systems. Processes related to foundations installation were adapted from [19]. The details of foundation installation activities can be found in **Table S2.** This paper also included the impacts of land-use transformation during foundation installation. The land-use impact was characterized by land transformation and land occupation. The coverage of one foundation and its scour protection vary from roughly 1195 square meters (m2) (type I), 291 m2 (type II), 763 m2 (type III), to 22 m2 (type IV) [2]. The land transformation is measured for the land cover change from one type to another. The land occupation measures how long a certain amount of area has been covered by one land cover type. Details are shown in **Table S3**.

Table S2: Marine activities related to installing one offshore wind foundation. Fuel rate = engine power (kW) × specific fuel consumption (g/kWh)/conversion factors (kg/l) × average load (%); HFO: Heavy fuel oil. Conversion factors (kg/l) for HFO: 1l = 0.983kg; conversion factors (kg/l) for diesel: 1l = 0.832kg

Foundation type[1]	Activity		Fuel, Equipment	# of equipment [19]	Work time (h) [19]	Fuel rate (l/h) [2]
I	Substrate clearance	Transport of excavator	HFO, Barge	1	72	100
		Dredging	Diesel, Excavator	1	72	0.455
		Disposal of substrate materials	HFO, Barge	1	70	100
	Substrate replacement	Transport of rock	HFO, Vessel	1	8.47	100
		Dumping of rock	HFO, Vessel	1	72	100
	Installation	Transport of foundation	Diesel, Tugboat	2	135	322.6
		Transport of jack-up	Diesel, Tugboat	2	1.8	322.6
		Construction of foundation	HFO, Jack-up vessel	1	24	170
	Scour protection	Transport of rock	HFO, Vessel	1	8.47	100
		Dumping of rock	HFO, Vessel	1	72	100
II	Driving pile	Transportation of pump/generator	HFO, Barge	1	24	100
		Injection of grout	Diesel, Pump/generator	1	24	185
	Installation	Tugboats for transport of foundations	Diesel, Tugboat	2	10.27	322.6
		Transport of jack-up	Diesel, Tugboat	2	3.6	322.6
		Construction of foundation	HFO, Jack-up vessel	1	24	170
	Scour protection	Transport of rock	HFO, Vessel	1	5.13	100
		Dumping of rock	HFO, Vessel	1	29	100
III	Driving pile	Transportation of pump/generator	HFO, Barge	1	24	100
		injection of grout	Diesel, Pump/generator	1	72	185
	Installation	Tugboats for transport of foundation	Diesel, Tugboat	2	144	322.6
		Transport of jack-up	Diesel, Tugboat	2	144	322.6
		Construction of foundation	HFO, Jack-up vessel	1	72	170

IV	Mooring	Transport of suction caisson	Diesel, Tugboat	1	24	322.6
		Pump out water	Diesel, Pump/generator	1	24	185
	Installation	Tugboats for transport of foundation and ballast	Diesel, Tugboat	2	168	322.6
		Transport of jack-up	Diesel, Tugboat	2	144	322.6
		Construction of foundation	HFO, Jack-up vessel	1	24	170

Table S3: Activities related to land occupation and transformation during installation of one foundation [19].

Foundation type	Activity	Amount	Unit
I	Gravel, unspecified, at mine/CH U (stone bed, 2m depth per base)	1669.0	t
	Gravel, unspecified, at mine/CH U (scour protection)	1794.5	t
	Occupation, water bodies, artificial	35837.7	m2a(m2*year)
	Transformation, from sea and ocean	1194.6	m2
	Transformation, to water bodies, artificial	1194.6	m2
II	Gravel, unspecified, at mine/CH U (per base)	687.5	t
	Occupation, water bodies, artificial	8740.3	m2a(m2*year)
	Transformation, from sea and ocean	291.3	m2
	Transformation, to water bodies, artificial	291.3	m2
III	Occupation, water bodies, artificial	22902.2	m2a(m2*year)
	Transformation, from sea and ocean	763.4	m2
	Transformation, to water bodies, artificial	763.4	m2
IV	Concrete, normal, at plant (suction caission)	500.0	t
	Occupation, water bodies, artificial	651.9	m2a(m2*year)
	Transformation, from sea and ocean	21.7	m2
	Transformation, to water bodies, artificial	21.7	m2

2.3.2.2 Installation of turbine

The turbine installation activities mainly include marine transportation of components from the harbor to erection site and component assembly by jack-up vessel. The installation time (work time) is nowadays only marginally more efficient per turbine as methods and procedures to install that were learnt and already well managed are not necessarily valid with the large turbines [20]. Therefore, this paper assumed turbine installation time is stable towards 2040. The fuel consumption of these processes was calculated based on [21]. The details of turbine installation activities can be found in **Table S4**.

Table S4: Marine activities related to installing one offshore wind turbine.

Activity	Fuel, Equipment	# of equipment [21]	Work time (h) [21]	Fuel rate (l/h) [21]
Transport of jack-up	Diesel, Tugboat	2	48	322.6
Assembly of wind turbine	HFO, Jack-up vessel	1	24	170

2.3.2.3 Installation of transmission infrastructure

Installation of transmission includes installation of transformer, substations and cables. Installation of cables is related to laying the cabling and assembling contain processes [2]. Each process needs different equipment, which should be mobilized from where they are before beginning on-site operation and demobilized to where they are after finishing the work. Work time for these processes was collected from [22] (See **Table S5**).

2.3.3 Operations and maintenance (O&M)

O&M processes involve inspections and maintenance of the physical plant and systems are mostly dependent on turbine failure rates [23]. This paper considered preventative and corrective maintenance.

2.3.3.1 Preventative maintenance (scheduled)

Preventative maintenance (scheduled) consists of regular inspection of turbines, cables and substations. These processes were modeled based on vessel work time and fuel consumptions.

2.3.3.2 Corrective maintenance (unscheduled)

Corrective maintenance includes unscheduled inspection and repair of turbines, cables and substations (shown in **Table S6**). Generators and blades are two most vulnerable components of offshore wind turbines [24]. The biggest contributor to the failure cost for offshore wind turbines is the generator and gearbox (if any) major replacement in the nacelle [25]. Due to complex long-term working conditions, blades tend to experience many internal (e.g. the fatigue failure) and external (e.g. environmental conditions) damages [26]. Work time for replacement of blades was assumed the same as replacement of nacelle due to lacking of data. Replacement processes were modeled based on [2].

2.3.3.3 Maintenance strategy

According to A Guide to UK Offshore Wind Operations and Maintenance [23], workboats are the most economic option for near-shore sites while the support by helicopters (heli-support) is necessary for sites further from shore. Helicopter transport in cases where difficult weather conditions prevent access by workboats. Further, for offshore wind farms located in deep water, more helicopters are needed to support offshore wind farm O&M. Port-based workboats become the only practical option for cases. However, lacking detailed representations of different vessels involved makes assessments of these activities in LCAs tentative. 50% marine vessel and 50% Jack-up vessel were assumed to be used during replacement processes. 100 flight-hours per wind turbine along 25 years life time is reported in [9]. Thus, this paper assumed 4 flight-hours per year per turbine.

Table S5: Marine activities related to installing transmission infrastructures for one wind turbine.

Activity			Fuel, Equipment	Mobilization and demobilization time (h) [2]	Work time (h/km) [2]	Fuel rate (l/h) [2]
Transformer substation installation [19]			HFO, jack-up vessel		8	170
Route clearance	Pre-sweep route	Dredging	HFO, vessel	96	24	100
	Route clearance	Anchor Handler	HFO, vessel	96	3.24	100
In-field operation	Tie-in and installation	Cable laying	HFO, vessel with plough	384	24	572.9
		Support	Diesel, vessel	96	24	262.5
		Burial assistance	Diesel, vessel		24	262.5
	Scour protection	Transportation of rock	HFO, vessel		0.128	100
		Rock placement	HFO, vessel		2	100
Shore connection	Installation	Cable laying	HFO, vessel with plough		3.53	572.9
	Scour protection	Transportation of rock	HFO, vessel		0.128	100
		Rock placement	HFO, vessel		2	100
Shore landing	Installation	Cable laying	HFO, vessel with plough		1.6	572.9
		Support	Diesel, vessel		4	262.5
	Shore spread	Cable yarder with sled winch	Diesel, winch	24	4	0.455

Table S6: Marine activities related to O&M (per incident per turbine per year).

**Activity**		**Fuel, Equipment**	**Work time (h)** [2]	**Fuel rate (l/h)** [2]
Preventative maintenance	Regular inspection of turbines	Diesel, Vessel	60	262.5
	Regular inspection of cables	Diesel, Vessel	336	150
	Regular inspection of substations	Diesel, Vessel	180	262.5
Corrective maintenance	Irregular inspection and repair	Diesel, Vessel	0.48	262.5
		Helicopter [9]	4	83.1[27]
Corrective maintenance	Replacement of nacelle	HFO, 50% Vessel and 50% Jack-up vessel	48	100, 170
	Replacement of blades [2]	HFO, 50% Vessel and 50% Jack-up vessel	48	100, 170
	Replacement of small components	HFO, 50% Vessel and 50% Jack-up vessel	0.96	100, 170

2.3.4 Decommissioning

2.3.4.1 Decommissioning of turbines

Wind turbines should be entirely removed from the site and then dissembled onshore. A heavy lift vessel or dynamic positioning vessel will usually be used [28]. The procedure performed will depend on the size and weight of the turbine, and will determine the lifting capacity and vessel's deck space. The emissions of decommission processes are mainly related to transportation of decommissioned wind turbines. The details of turbine decommission marine activities can be found in **Table S7**.

2.3.4.2 Decommissioning of foundations

This paper assumed foundations will be decommissioned. However, deep foundations may be costly to remove and it may have severe impacts on marine environment. Normally, foundations can be kept in site and available for repowering (replacement of the existing turbines into more powerful ones). Foundation lifetime is longer than turbines with approximately 100 year [29]. When foundations reach EoLs, there are two removal options proposed: the complete removal and cutting from a certain depth below the mud line and leaving the rest in situ [28]. But these processes are out of the discussion of this paper. Details can be found in [28].

2.3.4.3 Decommissioning of transmission pieces

This paper assumed cables will be left in situ. On the electrical side, array and export cables (transmission cables) could last more than 40 years, and the transformers 35 years [21]. Submarine cables (both internal and external cables) are usually buried into depths of more than a meter below the seabed, which will not pose safety risks for marine users and have limited environmental or pollution impacts [28]. The complete removal is considered to cause substantial damage and disruption to the seabed given the extensive length of the cables [30].

Table S7: Marine activities related to decommissioning of one offshore wind turbine.

Activity	Fuel, Equipment	# of equipment [21]	Work time (h) [21]	Fuel rate (l/h) [21]
Transport of jack-up	Diesel, Tugboat	2	24	322.6
Assembly of wind turbine	HFO, Jack-up vessel	1	12	170

2.4 Life cycle impact assessment

Table S8: A list of impact categories. Impact categories that marked in bulk were considered most related to OWE.

ReCiPe Midpoint (H) V1.13	
**Name**	**Unit**
agricultural land occupation, ALOP	square meter - year
**climate change, GWP100**	kg CO2-Eq
fossil depletion, FDP	kg oil-Eq
freshwater ecotoxicity, FETPinf	kg 1,4-DC.
freshwater eutrophication, FEP	kg P-Eq 13
human toxicity, HTPinf	kg 1,4-DC.
ionising radiation, IRP_HE	kg U235-Eq
**marine ecotoxicity, METPinf**	kg 1,4-DC.
**marine eutrophication, MEP**	kg N-Eq 46
**metal depletion, MDP**	kg Fe-Eq
natural land transformation, NLTP	square meter
ozone depletion, ODPinf	kg CFC-11.

particulate matter formation, PMFP	kg PM10-Eq
photochemical oxidant formation, POFP	kg NMVOC-.
terrestrial acidification, TAP100	kg SO2-Eq
terrestrial ecotoxicity, TETPinf	kg 1,4-DC.
urban land occupation, ULOP	square meter-year
water depletion, WDP	m3 water-.

Climate change, marine ecotoxicity, marine eutrophication, and metal depletion were considered as the most relevant impact categories in this study. OWE is key to energy transition and considered as a promising renewable energy source to mitigate greenhouse gases emissions (GHGs). Climate change is a widely used impact category to represent GHGs. OWE is located over shallow open waters in the sea and moving further into deep waters. Marine ecotoxicity and marine eutrophication are two impact categories directly linked to marine environment. Metal depletion will likely be a concern as several metals (e.g. steel, copper, and aluminum) are required along with the large-scale expansion of OWE development. This study mainly focus on these four impact categories but the environmental impact results of other impact categories could be found in **Results** in **Supporting Information II**.

Table S9: An overview of parameters related to the impacts of EP by the OWE. ✓ (✗) indicate the parameters that are (not) directly related to life cycle stage or component.

**Parameters**	**Life cycle stage**				**Component**			
	Manufacturing	Installation	O&M	Decommissioning	Nacelle	Rotor	Tower	Foundation
Capacity factor (CF)	✓	✓	✓	✓	✓	✓	✓	✓
Lifetime	✓	✓	✓	✓	✓	✓	✓	✓
Nominal capacity (NC)	✓	✓	✓	✓	✓	✓	✓	✓
Turbine size	✓	✗	✓	✗	✓	✓	✓	✓
Distance from shore	✗	✓	✓	✓	✗	✗	✗	✗
Technology market shares	✓	✓	✓	✓	✓	✓	✓	✓
Maintenance times	✗	✗	✓	✗	✓	✓	✗	✗
Replacement rates	✗	✗	✓	✗	✓	✓	✗	✗
Transportation strategy	✗	✗	✓	✗	✗	✗	✗	✗

3. Results and discussion

3.1 Environmental impact intensity

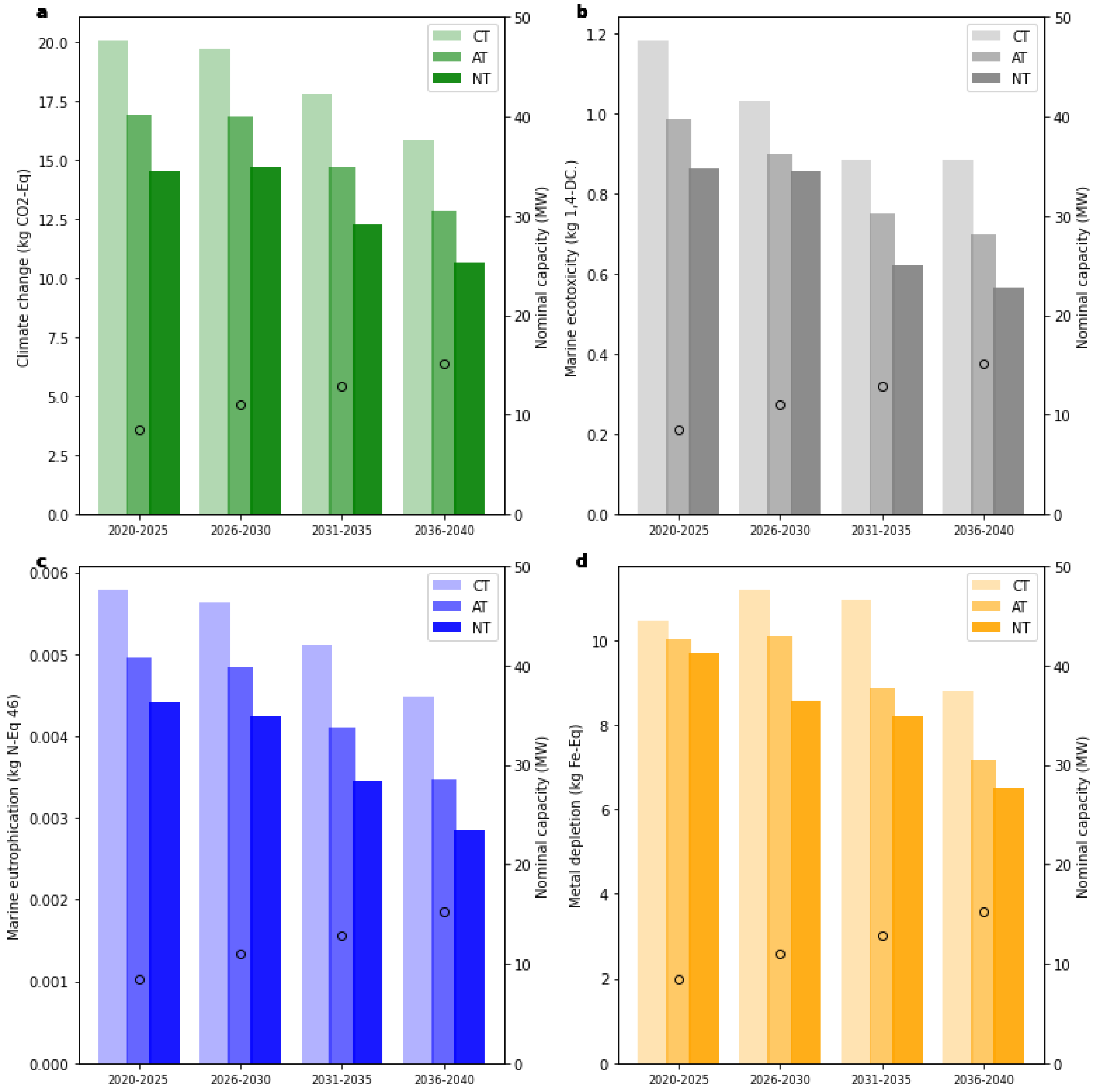

Figure S4: Environmental impacts per MWh (5-year average), under EoL_O recycling scenario. The bars from light color to dark color correspond to the impacts based on conventional technology (CT), advanced technology (AT), and new technology (NT) scenarios, respectively. The dots indicate the average nominal capacity at a given period.

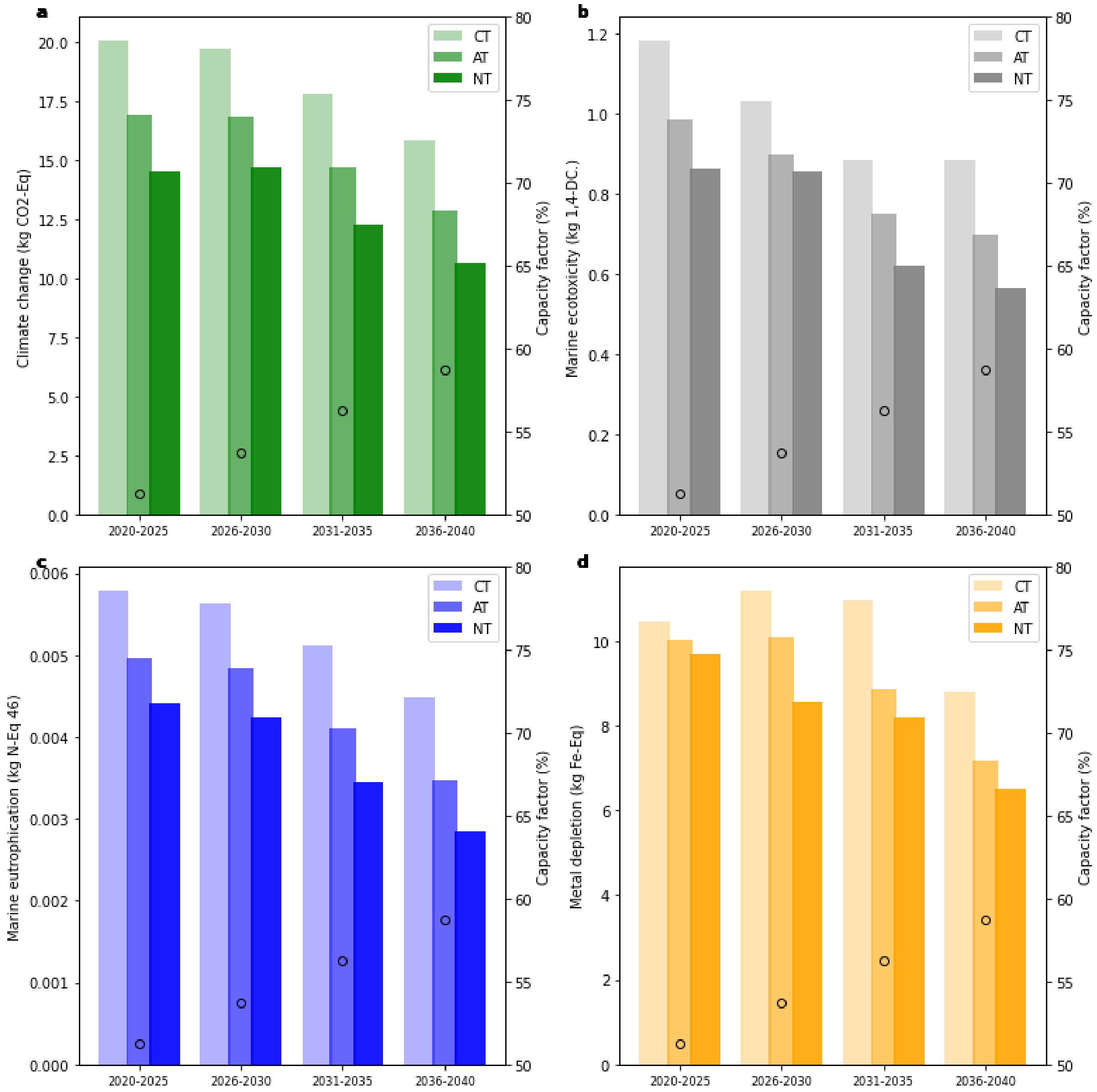

Figure S5: Environmental impacts per MWh (5-year average), under EoL_O recycling scenario. The bars from light color to dark color correspond to the impacts based on conventional technology (CT), advanced technology (AT), and new technology (NT) scenarios, respectively. The dots indicate the average capacity factor (CF) at a given period.

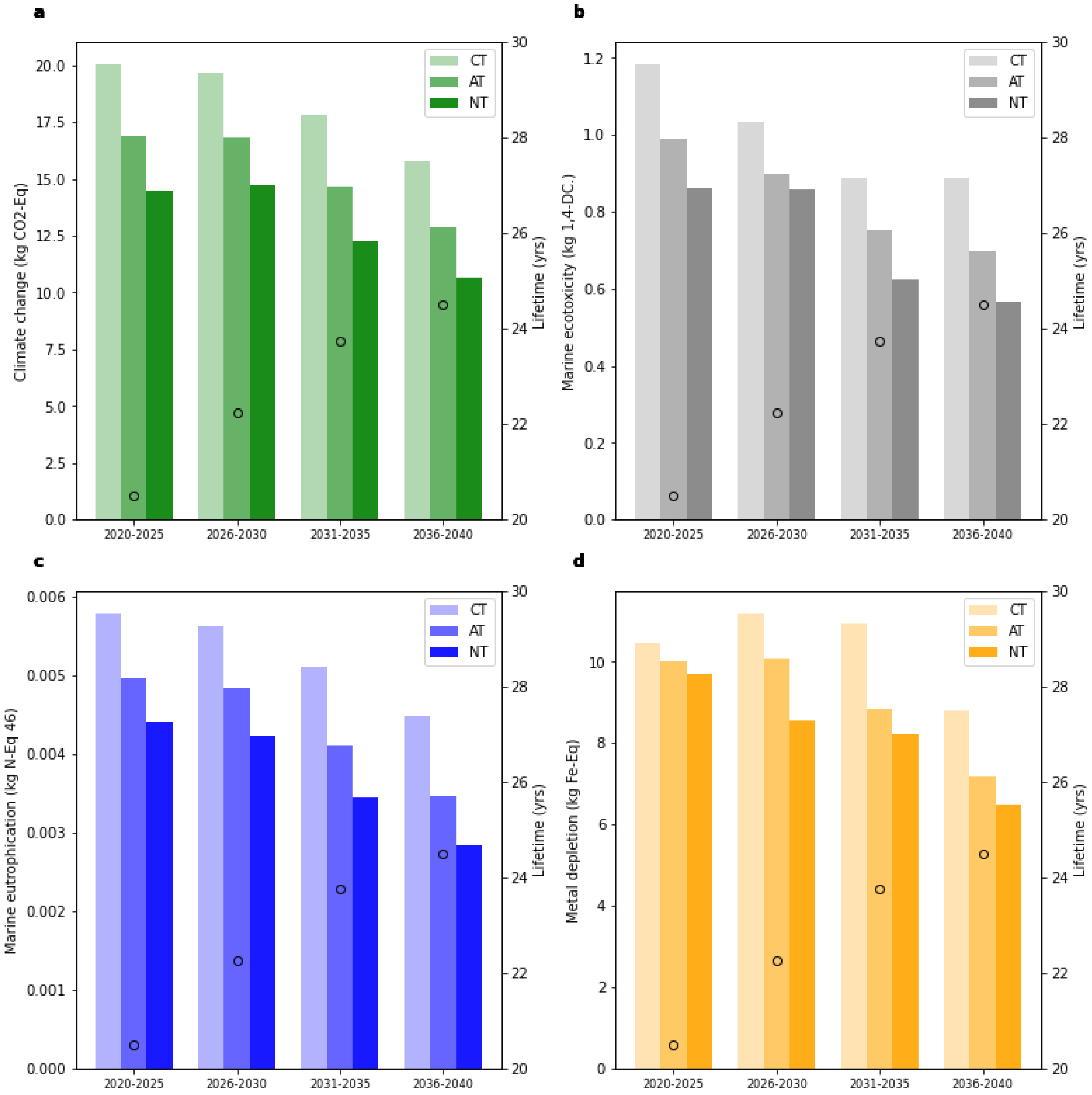

Figure S6: Environmental impacts per MWh (5-year average), under EoL_O recycling scenario. The bars from light color to dark color correspond to the impacts based on conventional technology (CT), advanced technology (AT), and new technology (NT) scenarios, respectively. The dots indicate the average lifetime at a given period.

The environmental impact intensities are influenced by several combined effects: **1)** increased lifetime leads to increased accumulative electricity production (**Figure S6**). The environmental impacts per MW (**Figure S7**) only slightly increase from 2020 to 2030 for all impact categories, which verifies the significance of lifetime extension on impact intensity decline; **2)** Nominal capacity will continue to increase in the future and leads to more powerful turbines, which have a larger rotor diameter corresponding to a high ratio of m2-of-swept-area-per-MW. According to **Figure S4**, climate change related GHG from 2030 to 2035, and from 2035 to 2040, is 2.0 (~13%) and 3.3 (~21%) kg CO2-eq./MWh lower than the average value from 2020 to 2030, respectively, when nominal capacity increases from 7.8 MW in 2020 to 15.6 MW in 2040 (twofold to 2020). **3)** Advanced and new technology development increase the capacity factor and further decrease the impact intensities (**Figure S5**).

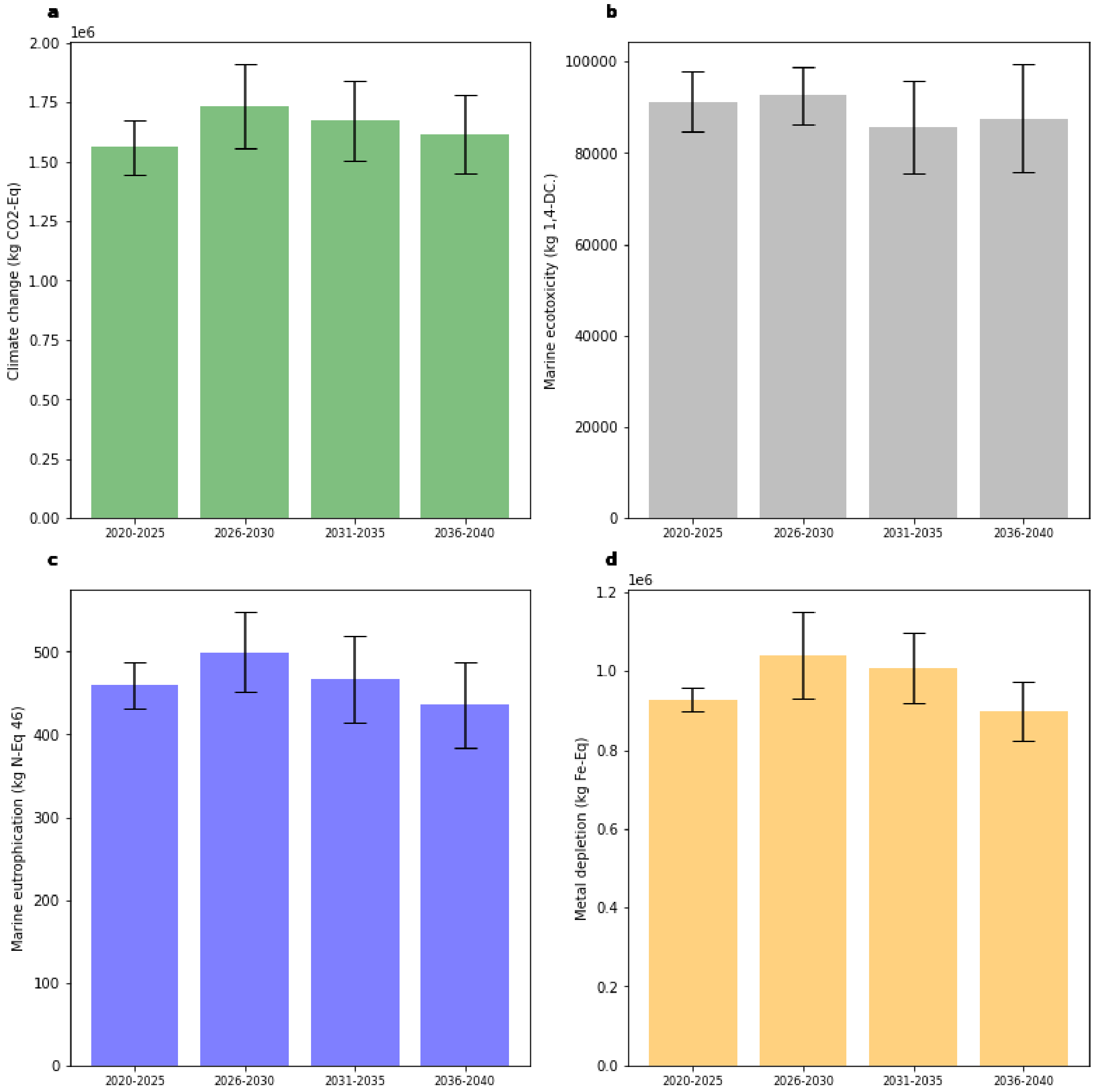

Figure S7: Environmental impacts per MW (5-year average). The main bars correspond to the values based on advanced technology (AT) scenario; the upper and lower bounds of error bars show the values based on conventional technology (CT) and new technology (NT) scenarios, respectively.

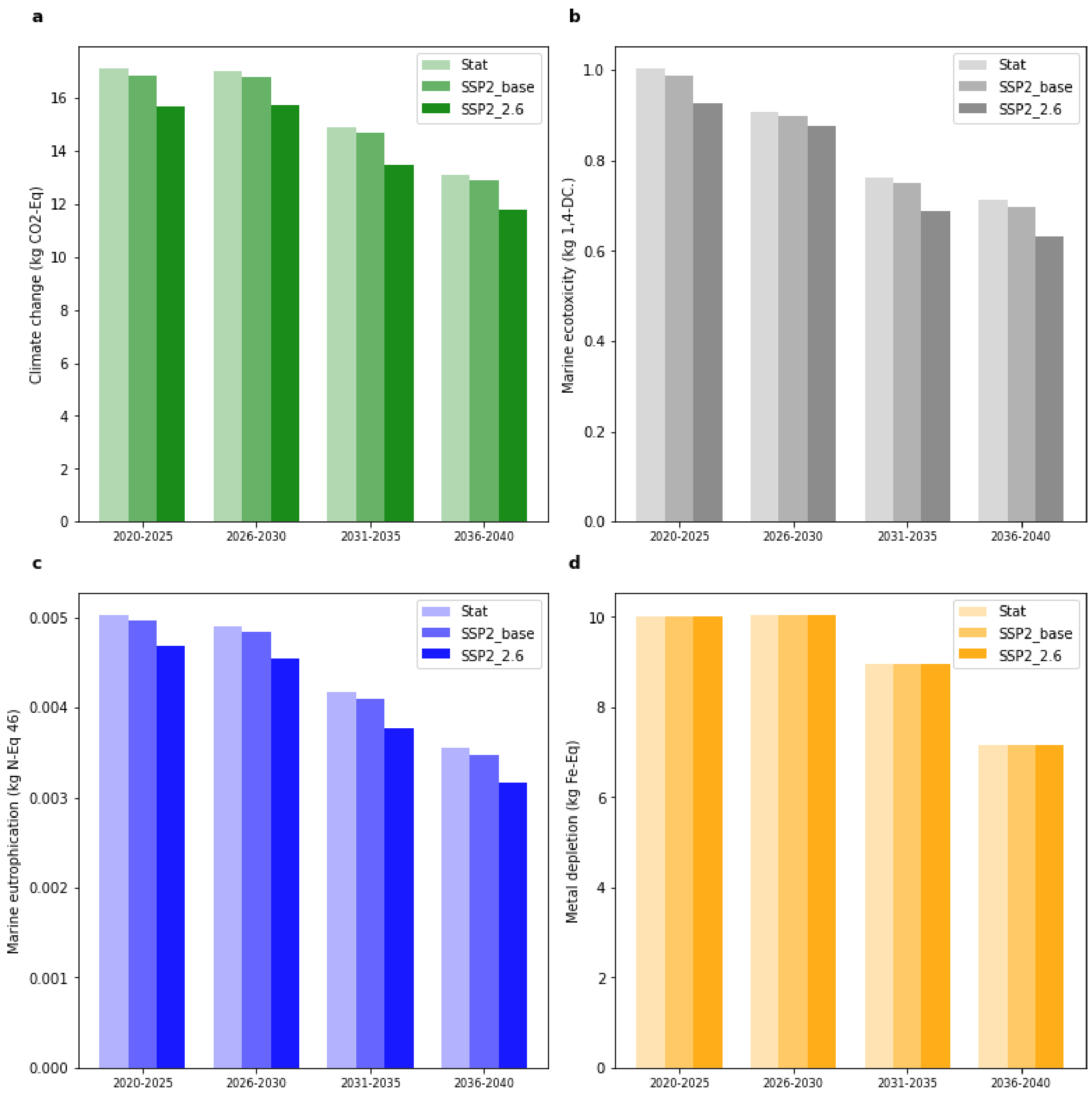

Figure S8: Comparative analysis on background scenarios, based on AT technology scenario and EoL_O recycling scenario. Stat: business as usual scenario (based on current background system, no changes); SSP2_base: Middle of the Road base scenario; SSP2_RCP2.6: Middle of the Road scenario that follows RCP2.6.

3.2 Fleet environmental impact

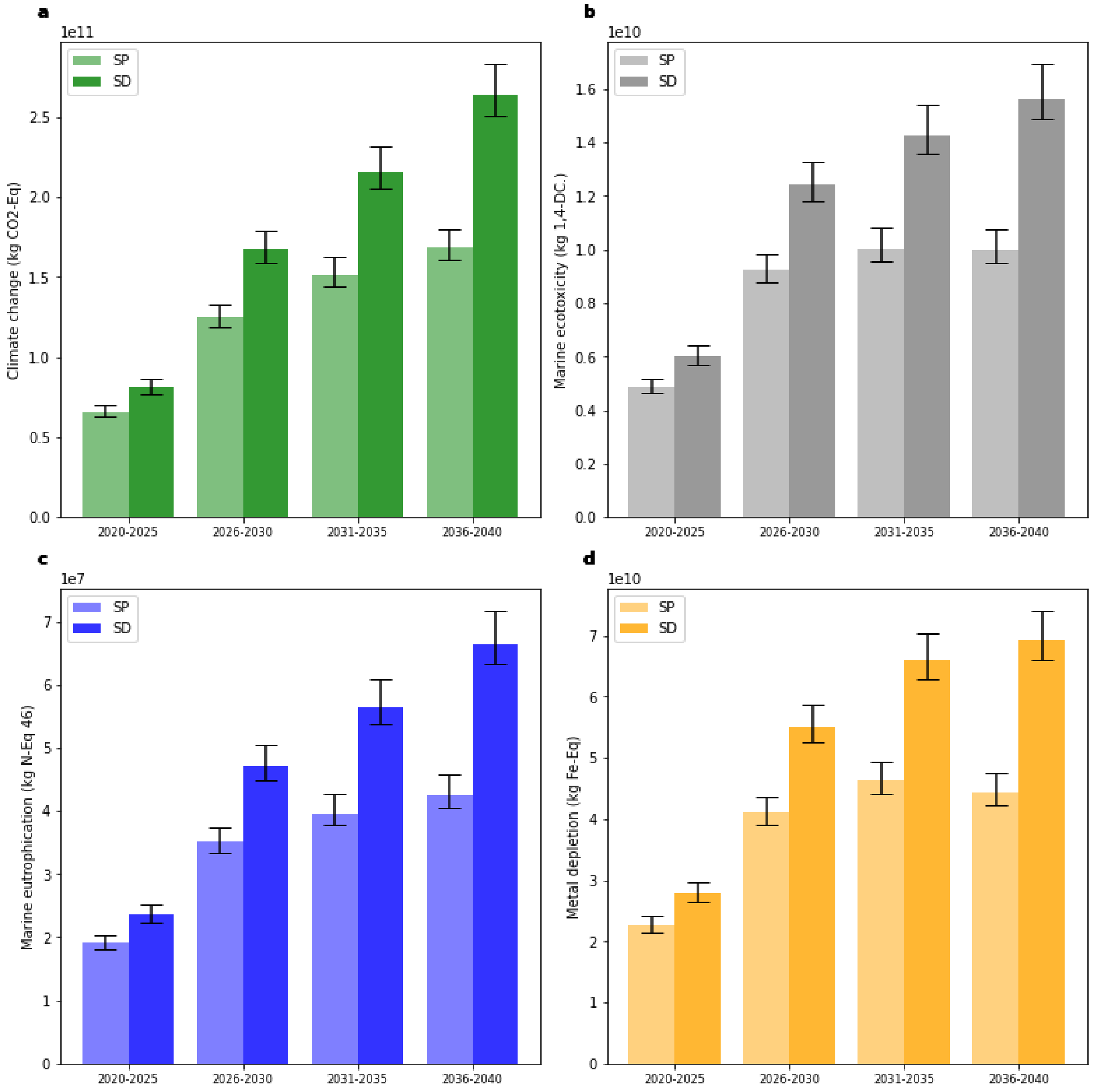

Figure S9: Total environmental impacts (5-year average), under EoL_O recycling scenario and SSP2-base background scenario. The bars with light and dark color represent the values based on stated policy (SP) and sustainable development (SD) capacity scenarios, respectively. The main bars correspond to the values based on advanced technology (AT) scenario; the upper and lower bounds of error bars show the values based on conventional technology (CT) and new technology (NT) scenarios, respectively. The current electricity mix was calculated based on the processes ''market group for electricity, high voltage'' in ecoinvent.

3.3 Contribution analysis

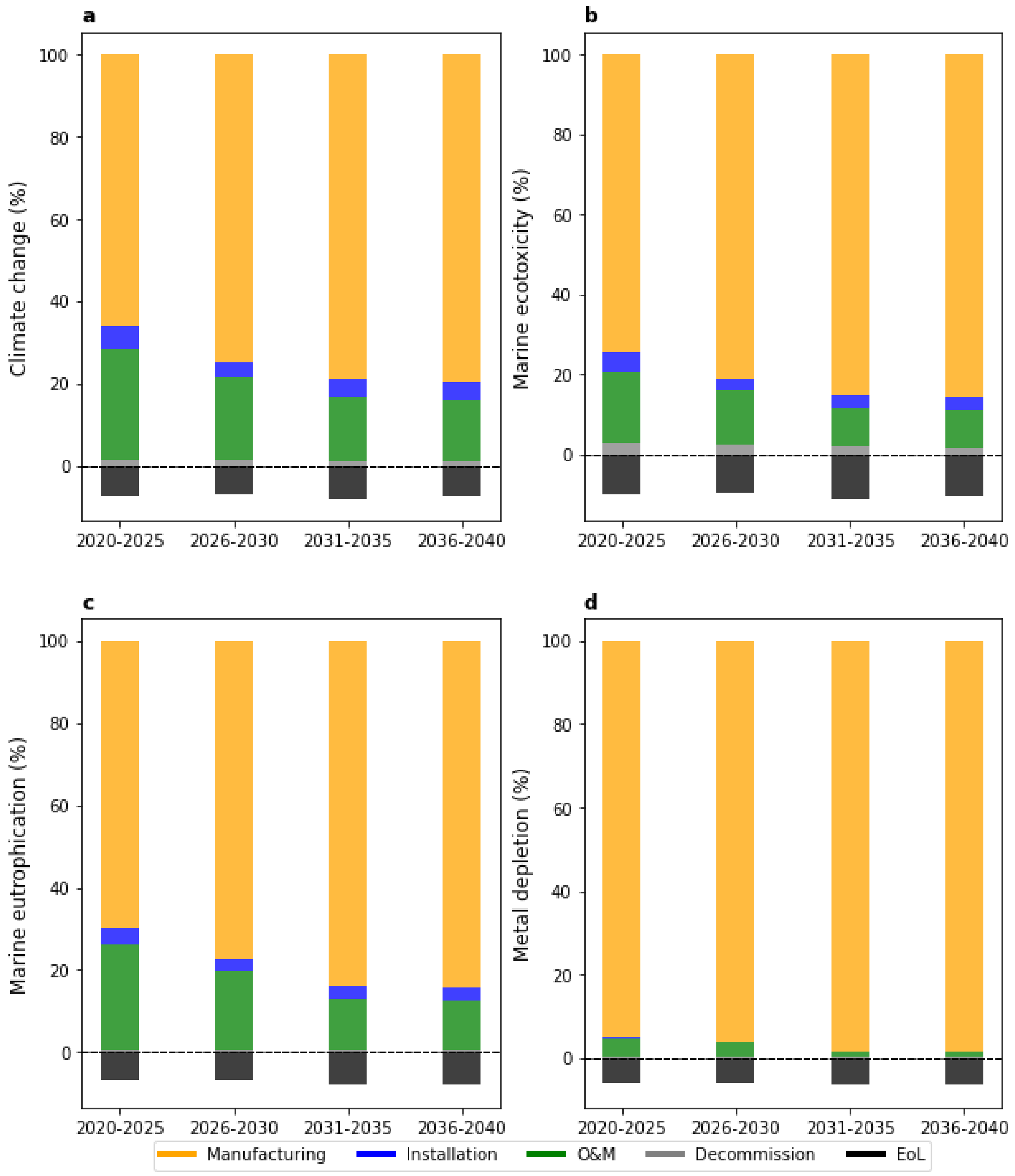

Figure S10: Contribution analysis on life cycle stage, i.e. manufacturing, installation, O&M, decommissioning, and EoL recycling, based on AT technology scenario, EoL_O recycling scenario, and SSP2-base background scenario. The black crosses show the percentages of impacts could be offset by EoL recycling.

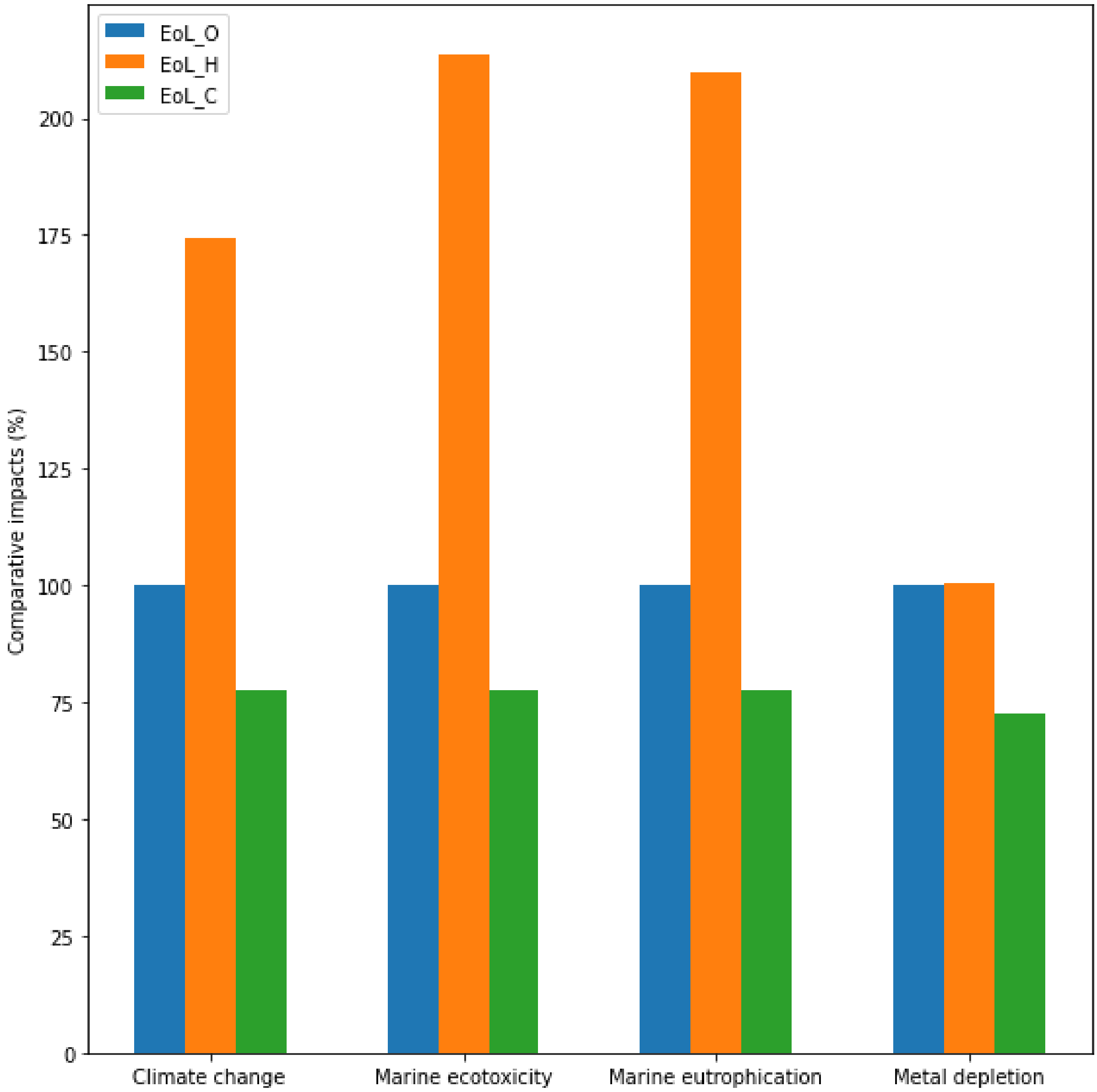

Figure S11: Comparison analysis on average (from 2020 to 2040) environmental impact reduction, under three EoL recycling scenarios, i.e. hypnotical EoL scenarios (EoL_H), optimistic EoL (EoL_O), and EoL conservative EoL (EoL_C), in terms of climate change, marine ecotoxicity, marine eutrophication, and metal depletion. Base EoL recycling scenario: EoL_O.

3.4 Sensitivity analysis

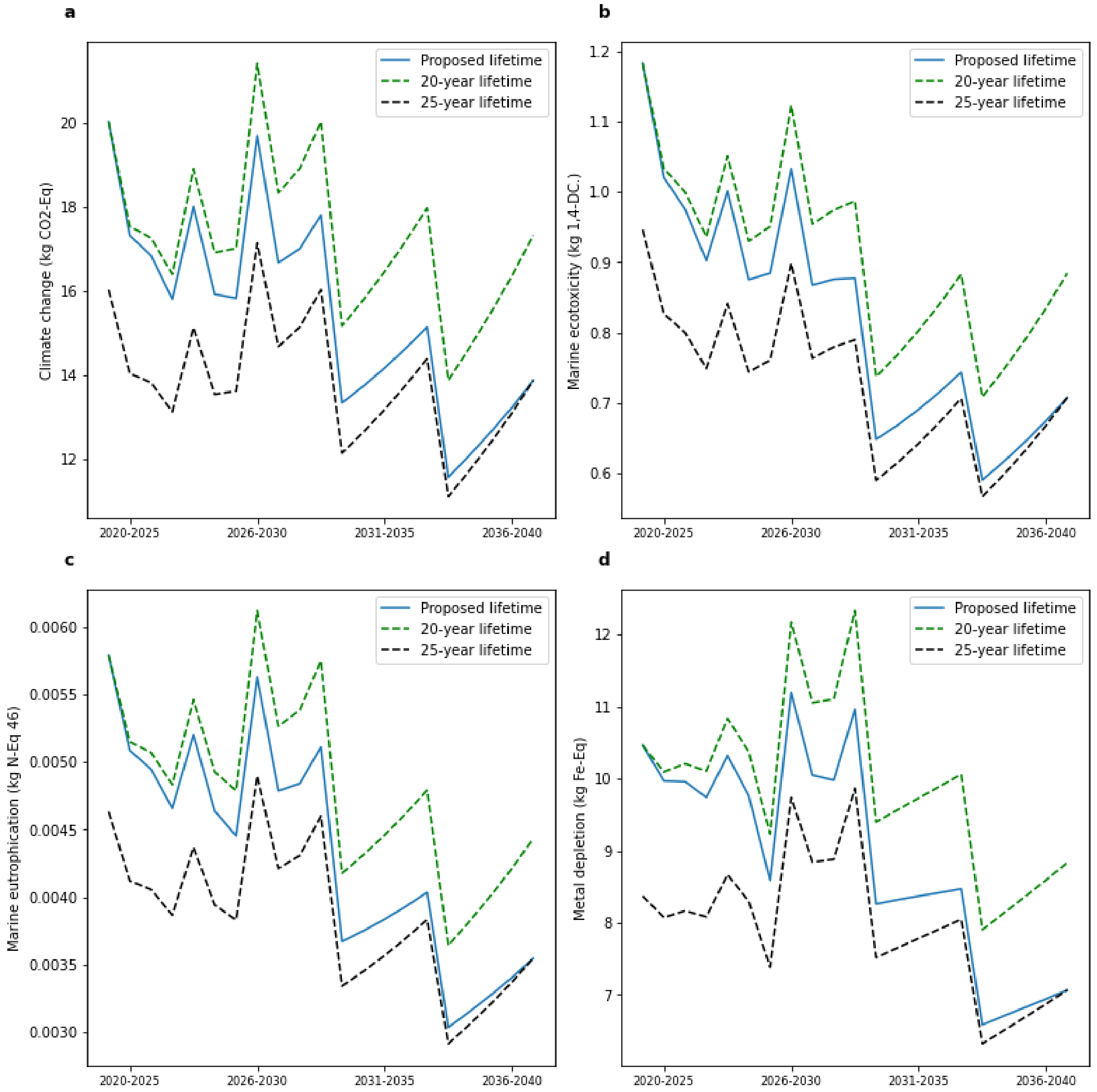

Figure S12: Sensitivity analysis on lifetime. Proposed lifetime indicates the lifetime assumed in this paper: dynamic lifetimes with a 20-year mean in 2020 that increases to a 25-year mean in 2040, and a 5-year standard deviation Normal distribution; A linear dynamic change was assumed for lifetimes from 2020 to 2040.

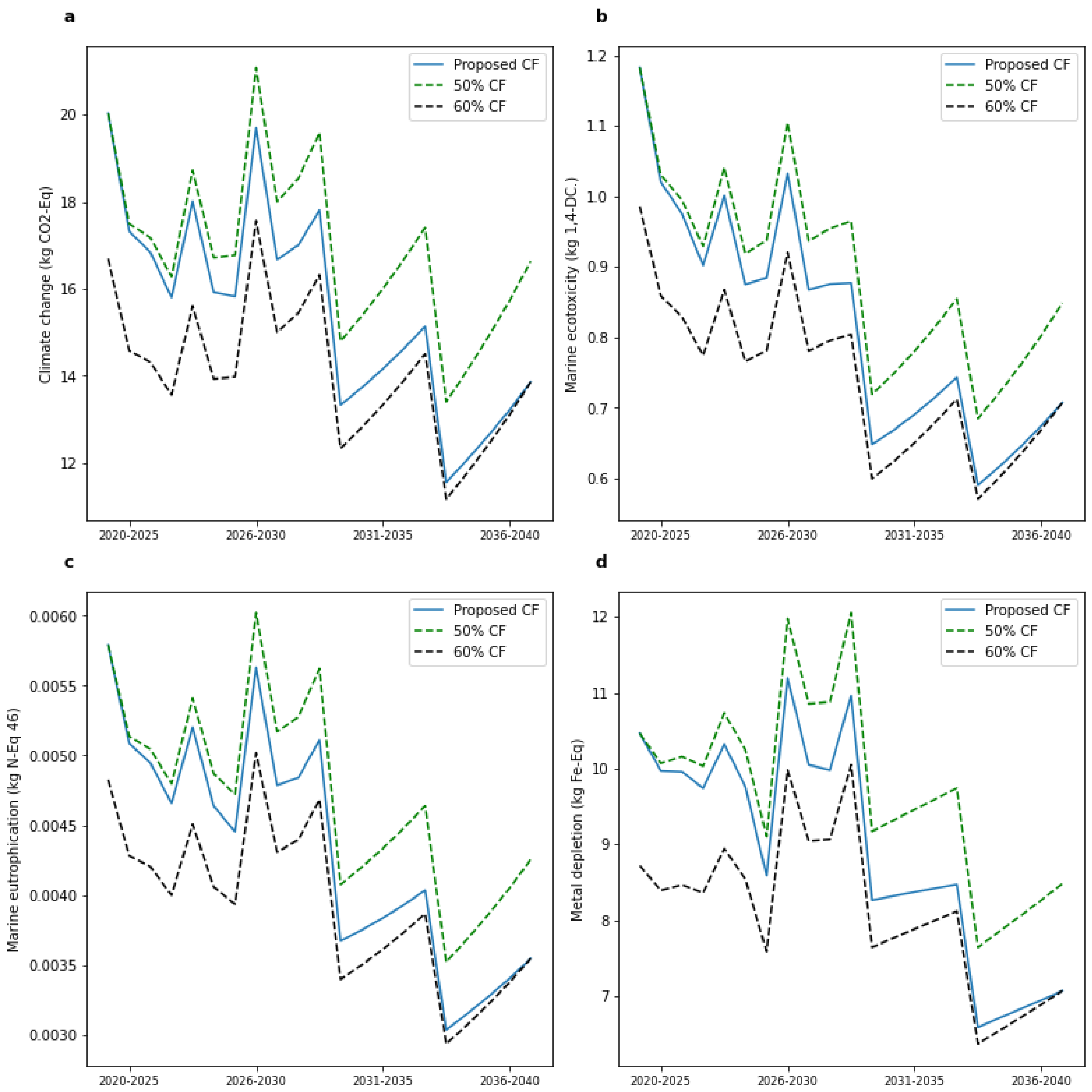

Figure S13: Sensitivity analysis on CF. Proposed CF indicates the lifetime assumed in this paper: 50% in 2020 and 60% in 2040, respectively. A linear dynamic change was assumed for CF from 2020 to 2040.

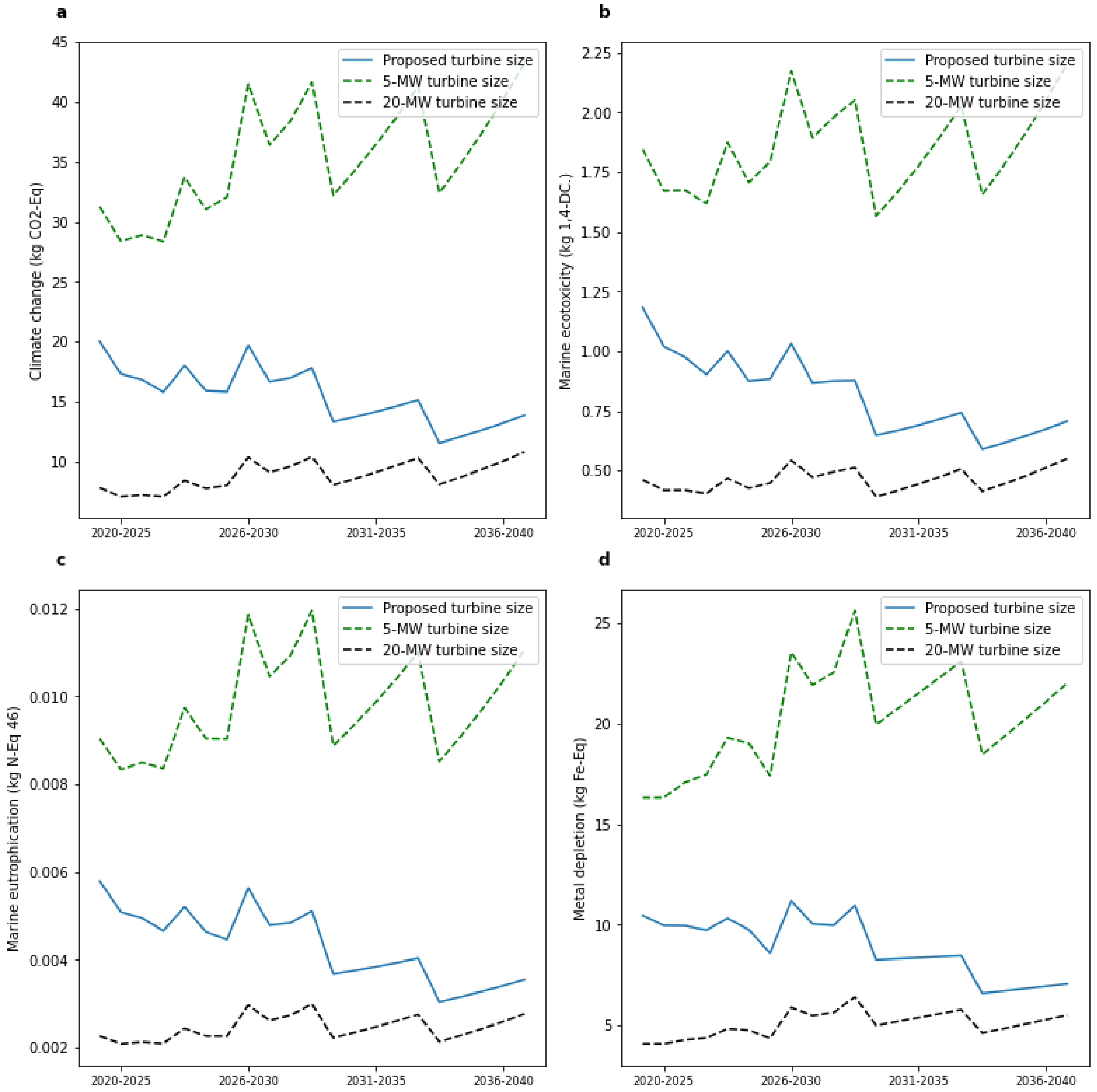

Figure S14: Sensitivity analysis on turbine size (nominal capacity). The proposed nominal capacity is estimated by the OLS regression based on projects from 4C offshore [15].

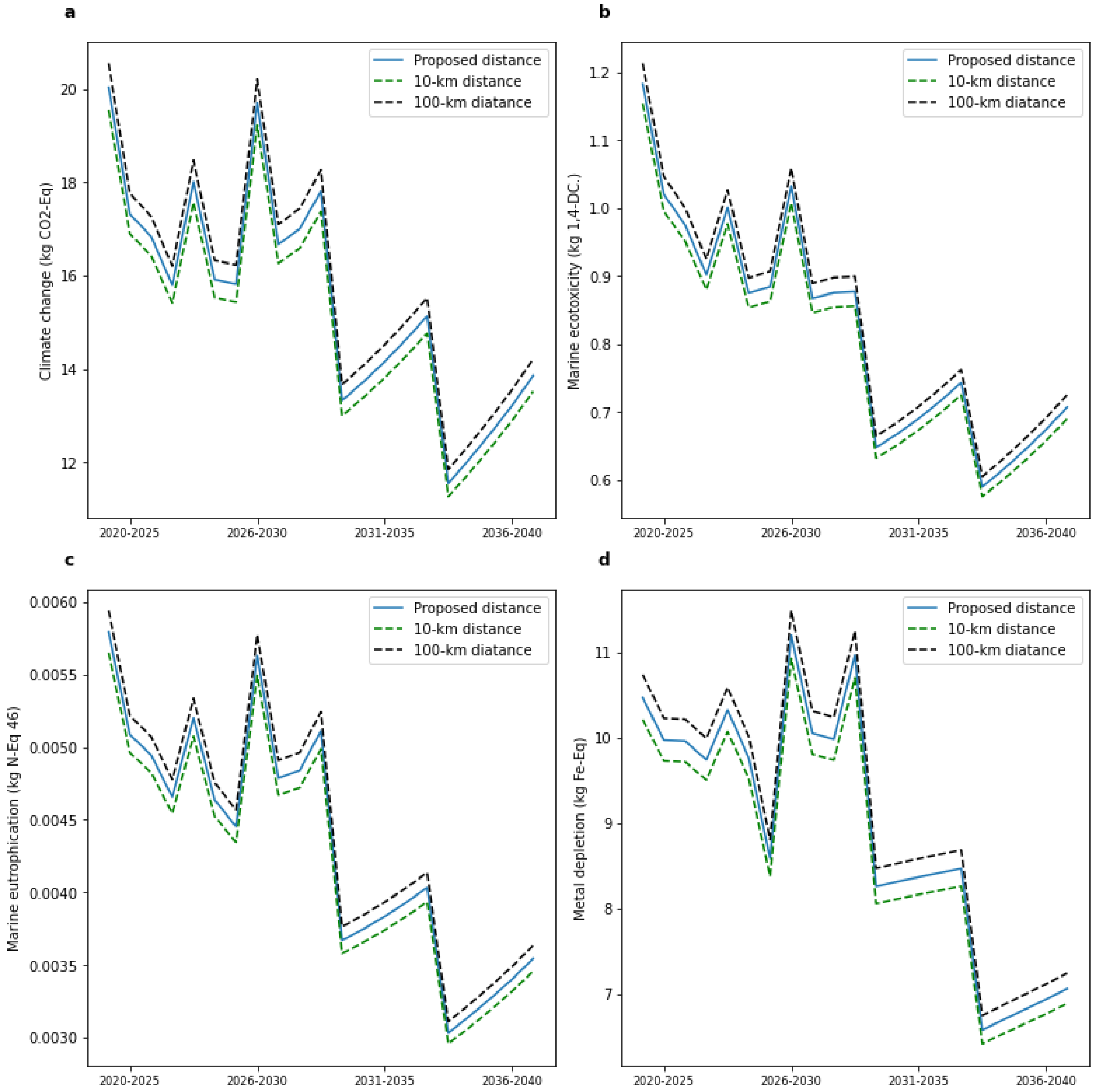

Figure S15: Sensitivity analysis on distance from shore. The proposed distance from shore was estimated by the OLS based on projects from 4C offshore [15].

3.5 Limitations and outlook

Marine transportation is normally modeled through theoretical considerations of energy use in transporting a mass over an assumed distance (tkm) [34]. OWE case is specific as most of the time that marine vessels and supporting equipment spend is at site, e.g. unloading components on top of the foundation. Specialized vessels are often required during transportation, uploading, and maintenance. In ecoinvent, barge, transoceanic freight ship, and port facilities are only represented for marine transportation [34]. This study modeled marine transportation based on the work time of vessels and supporting equipment, and associated fuel consumptions (**Table S2**, **S4, S5,** and **S6**). Currently, most offshore wind turbines are transported from harbor to site by tugboats [35] and installed by jack-up crane vessels in water depths up to 50m [36]. However, when wind turbines move further from shore with deep waters, specialized vessels are essential for transportation. Floating crane vessels are required to satisfy the high dynamic lifts of components for installing offshore wind

turbines in even deeper waters [37]. Moreover, larger turbine turbines in harsher environment will receive fatigue and corrosion damage, which require larger supporting infrastructures (e.g. specialized equipment and vessels) [32]. The availability of these infrastructures is a major challenge. There are numerous vessels in the small-scale market but more optimized vessels are still in the design phase. The scaling of background infrastructure and introduction of novel transportation technologies will likely further increase the impacts of installation, O&M, and decommissioning.

Table S10: Contribution analysis by the process, life cycle stage, component, and process in manufacturing and O&M, based on the AT scenario and EoL_O recycling scenario.

Impact category	Contributions						
	**Contributions by life cycle stage**						
Climate change	Manufacturing (~75%)	Installation (~5%)	O&M (~19%)	Decommissioning (~1%)	EoL (~-7%)		
Marine ecotoxicity	Manufacturing (~87%)	Installation (~3%)	O&M (~8%)	Decommissioning (~2%)	EoL (~-11%)		
Marine eutrophication	Manufacturing (~79%)	Installation (~4%)	O&M (~17%)	Decommissioning (~0%)	EoL (~-7%)		
Metal depletion	Manufacturing (~98%)	Installation (~0%)	O&M (~2%)	Decommissioning (~0%)	EoL (~-6%)		
	**Contributions by component**						
Climate change	Turbines (~56%)	Foundations (~41%)	Transmission (~3%)				
Marine ecotoxicity	Turbines (~64%)	Foundations (~34%)	Transmission (~2%)				
Marine eutrophication	Turbines (~57%)	Foundations (~40%)	Transmission (~3%)				
Metal depletion	Turbines (~77%)	Foundations (~19%)	Transmission (~4%)				
	**Contributions by process**						
Climate change	Reinforcing steel (~45%)	Diesel (~14%)	Carbon fiber (~11%)	Electricity (~6%)	Glass fiber (~5%)	Others (~19%)	
Marine ecotoxicity	Reinforcing steel (~43%)	Copper (~20%)	Low-alloyed steel (~10%)	Electronics (~7%)	Carbon fiber (~6%)	Others (~14%)	
Marine eutrophication	Reinforcing steel (~41%)	Glass fiber (~15%)	Diesel (~10%)	Carbon fiber (~8%)	Low-alloyed steel (~6%)	Electricity (~5%)	Others (~14%)
Metal depletion	Zinc (~52%)	Reinforcing steel (~21%)	Copper (~9%)	Low-alloyed steel (~7%)	Lead (~5%)	Others (~5%)	
	**Contributions by process in manufacturing**						
Climate change	Reinforcing steel (~60%)	Carbon fiber (~15%)	Glass fiber (~7%)	Electricity (~7%)	Low-alloyed steel (~5%)	Others (~6%)	
Marine ecotoxicity	Reinforcing steel (~49%)	Copper (~23%)	Low-alloyed steel (~11%)	Electronics (~6%)	Carbon fiber (~6%)	Others (~5%)	
Marine eutrophication	Reinforcing steel (~52%)	Glass fiber (~19%)	Carbon fiber (~10%)	Low-alloyed steel (~7%)	Electricity (~6%)	Others (~6%)	
Metal depletion	Zinc (~53%)	Reinforcing steel (~21%)	Copper (~9%)	Low-alloyed steel (~7%)	Lead (~5%)	Electronics (~3%)	Others (~2%)
	**Contributions by process in O&M**						
Climate change	Replacement of materials (~47%)	Diesel (~43%)	Heavy fuel oil (~10%)	Others (~0%)			
Marine ecotoxicity	Replacement of materials (~53%)	Diesel (~42%)	Others (~5%)				
Marine eutrophication	Replacement of materials (~52%)	Diesel (~37%)	Heavy fuel oil (~10%)	Others (~1%)			
Metal depletion	Replacement of materials (~99%)	Others (~0%)					

Table S11: Results of sensitivity analysis on embedded parameters, based on the AT technology scenario and EoL_O recycling scenario. Variation of impacts is calculated based on the cumulative impacts from 2020 to 2040.

**Parameters**		**Variation**	**Variation of climate change**	**Variation of marine ecotoxicity**	**Variation of marine eutrophication**	**Variation of metal depletion**
Technology market shares	PMSG-GB	+20%	+2.9%	+3.1%	+3.4%	+3.2%
	PMSG-GB	-20%	-2.3%	-2.3%	-2.3%	-2.3%
	PMSG-DD	+20%	+3.6%	+3.6%	+4.0%	3.8%
	PMSG-DD	-20%	-4.3%	-4.4%	-4.6%	-4.5%
	Semi-submersible	+20%	-9.4%	-9.1%	-9.5%	-9.3%
	Semi-submersible	-20%	+2.4%	+2.5%	+2.7%	+2.5%
Maintenance times		+20%	+0.7%	+0.7%	+0.9%	+0.8%
		-20%	-0.7%	-0.7%	-0.9%	-0.8%
Replacement rates		+20%	+1.3%	+1.3%	+1.5%	+1.4%
		-20%	-1.3%	-1.3%	-1.5%	-1.4%
Transportation strategy	Helicopter support	+20%	+0.5%	+0.5%	+0.6%	+0.6%
		-20%	-0.5%	-0.5%	-0.6%	-0.6%
Recycling rates		+20%	-4.0%	-4.0%	-4.4%	-4.1%
		-20%	+4.1%	+4.1%	+4.4%	+4.2%
Waste treatment	landfill processes	+20%	+2.0%	+2.1%	+2.2%	+2.2%
		-20%	-2.0%	-2.1%	-2.2%	-2.2%

Reference

(1) Wagner, H. J.; Baack, C.; Eickelkamp, T.; Epe, A.; Lohmann, J.; Troy, S. Life Cycle Assessment of the Offshore Wind Farm Alpha Ventus. *Energy* **2011**, *36* (5), 2459–2464. https://doi.org/10.1016/j.energy.2011.01.036.

(2) Tsai, L.; Kelly, J. C.; Simon, B. S.; Chalat, R. M.; Keoleian, G. A. Life Cycle Assessment of Offshore Wind Farm Siting: Effects of Locational Factors, Lake Depth, and Distance from Shore. *J. Ind. Ecol.* **2016**, *20* (6), 1370–1383. https://doi.org/10.1111/jiec.12400.

(3) Xie, J. bo; Fu, J. xun; Liu, S. yu; Hwang, W. sing. Assessments of Carbon Footprint and Energy Analysis of Three Wind Farms. *J. Clean. Prod.* **2020**, *254*, 120159. https://doi.org/10.1016/j.jclepro.2020.120159.

(4) Weinzettel, J.; Reenaas, M.; Solli, C.; Hertwich, E. G. Life Cycle Assessment of a Floating Offshore Wind Turbine. *Renew. Energy* **2009**, *34* (3), 742–747. https://doi.org/10.1016/j.renene.2008.04.004.

(5) Besseau, R.; Sacchi, R.; Blanc, I.; Pérez-López, P. Past, Present and Future Environmental Footprint of the Danish Wind Turbine Fleet with LCA_WIND_DK, an Online Interactive Platform. *Renew. Sustain. Energy Rev.* **2019**, *108* (April), 274–288. https://doi.org/10.1016/j.rser.2019.03.030.

(6) Reimers, B.; Özdirik, B.; Kaltschmitt, M. Greenhouse Gas Emissions from Electricity Generated by Offshore Wind Farms. *Renew. Energy* **2014**, *72*, 428–438. https://doi.org/10.1016/j.renene.2014.07.023.

(7) Bhandari, R.; Kumar, B.; Mayer, F. Life Cycle Greenhouse Gas Emission from Wind Farms in Reference to Turbine Sizes and Capacity Factors. *J. Clean. Prod.* **2020**, *277*, 123385. https://doi.org/10.1016/j.jclepro.2020.123385.

(8) Ma, C.; Bang, J.-I.; Tarantino, E.; Vela, A.; Yamane, D. Life Cycle Assessment of Greenhouse Gas Emissions for Floating Offshore Wind Energy in California. *Appl. Sci.* **2019**, No. May, 56.

(9) Arvesen, A.; Birkeland, C.; Hertwich, E. G. The Importance of Ships and Spare Parts in LCAs of Offshore Wind Power. *Environ. Sci. Technol.* **2013**, *47* (6), 2948–2956. https://doi.org/10.1021/es304509r.

(10) Cheng, M.; Zhu, Y. The State of the Art of Wind Energy Conversion Systems and Technologies: A Review. *Energy Convers. Manag.* **2014**, *88*, 332–347. https://doi.org/10.1016/j.enconman.2014.08.037.

(11) De Vries, E. *Wind Turbine Drive Systems: A Commercial Overview*; Woodhead Publishing Limited, 2013. https://doi.org/10.1533/9780857097491.2.139.

(12) Scott Semken, R.; Polikarpova, M.; Röyttä, P.; Alexandrova, J.; Pyrhönen, J.; Nerg, J.; Mikkola, A.; Backman, J. Direct-Drive Permanent Magnet Generators for High-Power Wind Turbines: Benefits and Limiting Factors. *IET Renew. Power Gener.* **2012**, *6* (1), 1–8. https://doi.org/10.1049/iet-rpg.2010.0191.

(13) Cooperman, A.; Eberle, A.; Lantz, E. Wind Turbine Blade Material in the United States: Quantities, Costs, and End-of-Life Options. *Resour. Conserv. Recycl.* **2021**, *168* (November 2020), 105439. https://doi.org/10.1016/j.resconrec.2021.105439.

(14) Li, Chen, José M. Mogollón, Arnold Tukker, Jianning Dong, Dominic von Terzi, Chunbo Zhang, and Bernhard Steubing. "Future material requirements for global sustainable offshore wind energy development." Renewable and Sustainable Energy Reviews 164 (2022): 112603.

(15) 4C Offshore. Global Offshore Wind Farms Database. 2013, p 4.

(16) (IEE), Fraunhofer Institute for Energy Economics and Energy System Technology.

(17) Baring-gould, I. Offshore Wind Plant Electrical Systems. **2014**.

(18) ABB. ABB Wind Turbine Converters PCS6000, Full Power Converter, up to 12 MW. **2019**, 12.

(19) Sustainable Society Consultant. **2012**.

(20) Fenu, B.; Attanasio, V.; Casalone, P.; Novo, R.; Cervelli, G.; Bonfanti, M.; Sirigu, S. A.; Bracco, G.; Mattiazzo, G. Analysis of a Gyroscopic-Stabilized Floating Offshore Hybrid Wind-Wave Platform. *J. Mar. Sci. Eng.* **2020**, *8* (6), 48–64. https://doi.org/10.3390/JMSE8060439.

(21) Birkeland, C. Assessing the Life Cycle Environmental Impacts of Offshore Wind Power Generation and Power Transmission in the North Sea. **2011**, No. June.

(22) Home | Van Oord. https://www.vanoord.com/en/ (accessed Sep 2021).

(23) GL Garrad Hassan. A Guide to UK Offshore Wind Operations and Maintenance. *Scottish Enterp. Crown Estate* **2013**, 42.

(24) Ren, Z.; Verma, A. S.; Li, Y.; Teuwen, J. J. E.; Jiang, Z. Offshore Wind Turbine Operations and Maintenance: A State-of-the-Art Review. *Renew. Sustain. Energy Rev.* **2021**, *144* (March). https://doi.org/10.1016/j.rser.2021.110886.

(25) Akay, B.; Ragni, D.; Ferreira, C. S.; Bussel, G. J. W. Van. Investigation of the Root Flow in a Horizontal Axis. *Wind Energy* **2013**, 1–20. https://doi.org/10.1002/we.

(26) Chou, J. S.; Chiu, C. K.; Huang, I. K.; Chi, K. N. Failure Analysis of Wind Turbine Blade under Critical Wind Loads. *Eng. Fail. Anal.* **2013**, *27*, 99–118. https://doi.org/10.1016/j.engfailanal.2012.08.002.

(27) Wernet, G.; Bauer, C.; Steubing, B.; Reinhard, J.; Moreno-Ruiz, E.; Weidema, B. The Ecoinvent Database Version 3 (Part I): Overview and Methodology. *Int. J. Life Cycle Assess.* **2016**, *21* (9), 1218–1230. https://doi.org/10.1007/s11367-016-1087-8.

(28) Topham, E.; McMillan, D. Sustainable Decommissioning of an Offshore Wind Farm. *Renew. Energy* **2017**, *102*, 470–480. https://doi.org/10.1016/j.renene.2016.10.066.

(29) Garcia, J. U. Estructuras de Apoyo En Energía Eólica Marica Retos y Requerimientos Tecnológicos Para El Año 2020 Oscar Yanguas Miñambres. **2012**.

(30) Smyth, K.; Christie, N.; Burdon, D.; Atkins, J. P.; Barnes, R.; Elliott, M. Renewables-to-Reefs? - Decommissioning Options for the Offshore Wind Power Industry. *Mar. Pollut. Bull.* **2015**, *90* (1–2), 247–258. https://doi.org/10.1016/j.marpolbul.2014.10.045.

(31) Liu, P.; Meng, F.; Barlow, C. Y. Wind Turbine Blade End-of-Life Options: An Eco-Audit Comparison. *J. Clean. Prod.* **2019**, *212*, 1268–1281. https://doi.org/10.1016/j.jclepro.2018.12.043.

(32) Jagadish, P. R.; Khalid, M.; Li, L. P.; Hajibeigy, M. T.; Amin, N.; Walvekar, R.; Chan, A. Cost Effective Thermoelectric Composites from Recycled Carbon Fibre: From Waste to Energy. *J. Clean. Prod.* **2018**, *195*, 1015–1025. https://doi.org/10.1016/j.jclepro.2018.05.238.

(33) The Crown Estate. Offshore Wind Operational Report 2015. *Gwec* **2015**, No. December.

(34) Linington, P. Transport Service. *Comput. Commun.* **1980**, *3* (5), 202–207. https://doi.org/10.1016/0140-3664(80)90150-4.

(35) Arvesen, A.; Birkeland, C.; Hertwich, E. G. The Importance of Ships and Spare Parts in LCAs of Offshore Wind Power. *Environ. Sci. Technol.* **2013**, *47* (6), 2948–2956. https://doi.org/10.1021/es304509r.

(36) Faculty, T.; Science, E.; Technology, M. *Wilson Ivan Guachamin Acero Assessment of Marine Opera- Tions for Offshore Wind Turbine Installation with Emphasis on Response-Based Operational Lim- Its*; 2016.

(37) Verma, A. S.; Jiang, Z.; Ren, Z.; Gao, Z.; Vedvik, N. P. Response-Based Assessment of Operational Limits for Mating Blades on Monopile-Type Offshore Wind Turbines. *Energies* **2019**, *12* (10), 1–26. https://doi.org/10.3390/en12101867.
